# Population-Scale Polymorphic Short Tandem Repeat Provides an Alternative Strategy for Allele Mining in Cotton

**DOI:** 10.3389/fpls.2022.916830

**Published:** 2022-05-06

**Authors:** Huan Mei, Ting Zhao, Zeyu Dong, Jin Han, Biyu Xu, Rui Chen, Jun Zhang, Juncheng Zhang, Yan Hu, Tianzhen Zhang, Lei Fang

**Affiliations:** ^1^Zhejiang Provincial Key Laboratory of Crop Genetic Resources, Institute of Crop Science, Plant Precision Breeding Academy, College of Agriculture and Biotechnology, Zhejiang University, Hangzhou, China; ^2^Hainan Institute of Zhejiang University, Sanya, China

**Keywords:** *Gossypium hirsutum*, short tandem repeats, polymorphism, STR-GWAS, agronomic traits

## Abstract

Short tandem repeats (STRs), which vary in size due to featuring variable numbers of repeat units, are present throughout most eukaryotic genomes. To date, few population-scale studies identifying STRs have been reported for crops. Here, we constructed a high-density polymorphic STR map by investigating polymorphic STRs from 911 *Gossypium hirsutum* accessions. In total, we identified 556,426 polymorphic STRs with an average length of 21.1 bp, of which 69.08% were biallelic. Moreover, 7,718 (1.39%) were identified in the exons of 6,021 genes, which were significantly enriched in transcription, ribosome biogenesis, and signal transduction. Only 5.88% of those exonic STRs altered open reading frames, of which 97.16% were trinucleotide. An alternative strategy STR-GWAS analysis revealed that 824 STRs were significantly associated with agronomic traits, including 491 novel alleles that undetectable by previous SNP-GWAS methods. For instance, a novel polymorphic STR consisting of GAACCA repeats was identified in *GH_D06G1697*, with its (GAACCA)_5_ allele increasing fiber length by 1.96–4.83% relative to the (GAACCA)_4_ allele. The database CottonSTRDB was further developed to facilitate use of STR datasets in breeding programs. Our study provides functional roles for STRs in influencing complex traits, an alternative strategy STR-GWAS for allele mining, and a database serving the cotton community as a valuable resource.

## Introduction

Short tandem repeats (STRs) are repetitive genome sequence elements that range in length from 1 to 10 bp (Hannan, 2010) and are widely present in most eukaryotic, prokaryotic, and viral genomes across the tree of life (Oliveira et al., 2006; Torresen et al., 2019). STRs can regulate phenotypes involved in dozens of human diseases, such as Huntington’s disease (Duyao et al., 1993), neuronal intranuclear inclusion disease (Tian et al., 2019), autism (Trost et al., 2020), and Friedreich ataxia (Montermini et al., 1997). In plants, STRs regulate prominent biological processes such as development and stress response. For example, in *Arabidopsis thaliana*, a STR in *PFT1* (*AT1G25540*) has been shown to regulate flowering in a photoperiod-dependent manner (Rival et al., 2014), while another STR with dramatically expanded TTC/GAA repeat in the intron of *IIL1* (*AT4G13430*) causes an environment-dependent reduction in IIL1 activity and severely impairs growth (Sureshkumar et al., 2009). In chickpeas, a STR with CT repeat length variation in the 5′-UTR of the *CaIMP* gene might regulate phytic acid levels to confer drought tolerance in natural populations (Joshi-Saha and Reddy, 2015). In cotton, STR length variations in invertases (EC 3.2.1.26) were found to be involved in fiber development (Taliercio et al., 2010). These observations support the importance of STRs as a reservoir of functional genetic variation.

Next-generation sequencing (NGS) technologies have generated massive amounts of genomic data with high coverage and depth (Pareek et al., 2011; van Dijk et al., 2014), which also contains a great deal of information on polymorphic STRs. However, genomic studies have mainly focused on SNPs and indels, and only to a lesser extent on STRs (Fang et al., 2017; Ma et al., 2018). Recent STR-GWAS publications have revealed additional genetic associations of STRs with traits including height, serum urea, and hair phenotypes in human (Sun et al., 2012; Gymrek et al., 2016; Mukamel et al., 2021) and flowering in *A. thaliana* (Press et al., 2018; Reinar et al., 2021). Another study reported that length variations had more impact on phenotype than SNP variations (Huang et al., 2015). STRs can impact phenotype through several mechanisms: modulating the binding of transcription factors (TFs), changing the spacing of regulatory sites (Willems et al., 1990), altering splicing efficiency (Hefferon et al., 2004), and altering the structure of proteins (La Spada et al., 1991). In addition to their biological importance, STRs are of substantial value as molecular markers, being both highly abundant and highly polymorphic (Paques et al., 1998; Saha et al., 2003). STRs are generated by DNA-polymerase strand slippage during replication and recombination events, which results in the addition or deletion of repeat units (Paques et al., 1998; Verstrepen et al., 2005; Fan and Chu, 2007); this origin confers higher mutation rates than the average for other mutations, ranging from 10^−3^ to 10^−6^ per generation (Ellegren, 2004; Sun et al., 2012). Consequently, STRs have seen wide use for genetic applications such as map construction, quantitative trait loci (QTL) mapping, genotype fingerprinting, and genetic diversity analyses (Kalia et al., 2011).

Cotton (*Gossypium* spp.) is one of the most important natural textile fiber and oil crops around the world. In keeping with that status, more than 3,000 natural accessions and commercial cultivars have been sequenced to date (Fang et al., 2017, 2021; Wang et al., 2017; Ma et al., 2018, 2021b; He et al., 2021; Yuan et al., 2021). However, identification of STRs has so far been limited to two single individuals constituting the reference genome sequences of *G. hirsutum* (*Gh*) acc. TM-1 and *G. barbadense* (*Gb*) cv. Hai7124 (Blenda et al., 2006; Wang et al., 2015; Wu et al., 2020). Cotton STRs and their genotyping remain largely unknown at the population level, and few functional STRs have yet been reported. Here, we constructed a high-density STR map for *G. hirsutum* using WGS data from 911 cultivars (Fang et al., 2017, 2021; Ma et al., 2018) and evaluated in detail the potential impacts of STRs on cotton gene structure and agronomic traits.

## Materials and Methods

### Sources of Genomic Sequences and Phenotype Data

WGS data of 911 cotton accessions were downloaded from the National Center for Biotechnology Information (NCBI) database. These originated from three independent panels of GWAS projects: PRJNA375965, containing 258 global modern improved cultivars or enhanced lines (termed P1; Fang et al., 2017); PRJNA613140, containing 234 commercial cultivars (termed P2; Fang et al., 2021); and SRP115740, containing 419 worldwide accessions (termed P3; Ma et al., 2018). The corresponding phenotype data, including measures of quality (FE, FL, FM, FS, LU, MAT, SCI, and FU), yield (BW, FWBP, LI, LP, SI, and BN), and other traits (VW and FD), were, respectively, retrieved from http://mascotton.njau.edu.cn/info/1058/1132.htm (Fang et al., 2017), http://cotton.zju.edu.cn/ (Fang et al., 2021), and http://cotton.hebau.edu.cn/zlxz.html (Ma et al., 2018). The data of PP and PH are new in this study, which were provided in Supplementary Table S1.

### Identification of Polymorphic STRs

In total, we obtained 8.76 Tb of raw sequences for 911 samples. The WGS data were pre-processed using fastp (v 0.12.2) with default parameters to remove adapters and low-quality bases (Chen et al., 2018). The clean data were aligned against the genetic standard line of *Gh* TM-1 (V 2.1; Hu et al., 2019)^1^ using Burrows-Wheeler Aligner with the mem model (Li and Durbin, 2009). Mapping results were then converted into the BAM format and sorted using samtools (v 1.6; Li et al., 2009). Duplicate reads were removed using Picard (v 1.124).^2^ After mapping, we performed STR calling separately for the three sets of samples using HipSTR v.0.5, which takes aligned BAM files as input and returns the maximum likelihood diploid STR allele sequences for each sample. Samples were genotyped separately with nondefault parameters (--minreads 5, --max-str-len 300, and -def-stutter-model; Willems et al., 2017). This workflow allowed us to obtain a high-density polymorphic STR map comprising 556,426 STRs.

To study the localization of STRs with protein coding genes, we used the GFF annotation files of TM-1 (V 2.1; Hu et al., 2019) to measure distance to the nearest gene TSS, and additionally categorized variants based on their overlap with introns, exons, promoters, and transposable elements (TE > 500 bp). Overlapping was performed using the intersectBed tool of the BEDTools suite (v.2.28.0; Quinlan and Hall, 2010). For each STR, we defined the canonical repeat unit intergrade with EST-SSR markersMIcroSAtellite (MISA; Thiel et al., 2003)^3^ with basic motifs from mono- to decanucleotide. Minimum repeat count for each motif length was set as 6, 3, 2, 2, 2, 2, 2, 2, 2, and 2, respectively. STRs were further annotated using ANNOVAR (2016-02-01; Wang et al., 2010) and categorized as being sited in either exonic or intergenic regions. Genes with exonic STRs were then classified according to whether the STRs were ORF-disrupting.

### Gene Ontology and KEGG Enrichment Analysis

Gene Ontology (GO) analysis of 6,021 genes with exonic STRs was performed using the R package Gostats (Falcon and Gentleman, 2007). All genes in the cotton genome were used as background. The GO annotation for cotton was obtained from TM-1 v2.1 (Hu et al., 2019). To reduce redundancy of GO terms, significant GO terms were clustered according to similarity using the R package *simplifyEnrichment* (v 1.0.0).^4^ Kyoto Encyclopedia of Genes and Genomes (KEGG) enrichment analysis was conducted using TBtools (v1.0971; Chen et al., 2020). GO terms exhibiting a corrected (after false discovery rate adjustment) *p* ≤ 0.05 were considered to be significantly enriched.

To identify STR-associated genes related to flowering, we retrieved *Arabidopsis* flowering genes from FLOR-ID, which contains information on 306 genes and links to 1,595 publications gathering the work of >4,500 authors (Bouche et al., 2016). We then identified 4,562 flowering genes in cotton using BLAST and homology to the set of *A. thaliana* flowering genes (Supplementary Table S2).

### Gene Expression Analysis

A total of 65 accessions were collected from the Institute of Cotton Research at CAAS, the core germplasm samples were previously genotyped by our laboratory (Fang et al., 2017). Plants were grown in a farm environment during the summer of 2018 at Dangtu, Anhui, China. Two independent biological samples of each accession were grown in different experimental fields. For fiber collection, 16–18 plants were grown per accession; the collected 20-DPA fibers were bulked for total RNA extraction and sequencing. Clean RNA-seq reads (150 bp paired-end) were aligned to the *Gh* TM-1 v2.1 reference genome using Hisat2 (V 2.1.0) with parameter (--dta; Pertea et al., 2016). Mapped reads in each library were subsequently passed to StringTie (V 2.0) for transcript assembly (Pertea et al., 2016) using annotated TM-1 transcripts as the reference transcriptome. The obtained expression values were normalized to fragments per kilobase of exon model per million mapped fragments (FPKM).

### Genome-Wide Association Analysis

Association analyses were conducted on the P1, P2, and P3 panels collected from three published GWAS (Fang et al., 2017, 2021; Ma et al., 2018). For the present analysis, we utilized high-quality STRs [minor allele frequency (MAF) > 0.05, missing ratio <30%, and biallelic]. VCFs were filtered using vcftools (v 0.1.13; Danecek et al., 2011), leaving 14,241 STRs for P1 (Fang et al., 2017), 8,504 STRs for P2 (Fang et al., 2021), and 36,557 STRs for P3 (Ma et al., 2018).

Association analysis was performed with a linear mixed model through Efficient Mixed-Model Association eXpedited (EMMAx, emmax-beta-07Mar2010; Kang et al., 2010). The kinship matrix was calculated with a centered identity-by-state (IBS) matrix. The genome-wide significant *p*-value thresholds were set following the adjusted Bonferroni method for multiple testing correction, *p* < 1/*N*, where *N* is the number of STRs used for GWAS; for the three panel populations, this yielded respective thresholds of *p* < 7.02 × 10^−5^ for P1 (Fang et al., 2017), *p* < 1.18 × 10^−4^ for P2 (Fang et al., 2021), and *p* < 2.74 × 10^−5^ for P3 (Ma et al., 2018). To identify novel trait-associated loci, we conducted a SNP-GWAS using similar methodology for comparison. Pairwise linkage disequilibrium and r values between STRs were calculated by PLINK software. STR-associated loci for which no trait-associated SNPs were in linkage disequilibrium (LD, *r* > 0.1) were defined as Novel Significant association loci (termed novel STRs).

### DNA Extraction and STR Amplification

Cotton genomic DNA was extracted from young leaves using a modified cetyltrimethylammonium bromide (CTAB) method (Paterson et al., 1993). Amplification system: 50 μl reaction volume comprising 25 μl of 2× Rapid Taq Master Mix, 10 μmol L^−1^ STR primer, and 100 ng DNA template. Amplification procedure: pre-denaturation at 95°C for 5 min; denaturation at 95°C for 15 s, annealing at 53°C for 15 s, and extension at 72°C for 15 s, repeated for a total of 34 cycles; and finally, extension at 72°C for 5 min. Polymerase chain reaction (PCR) primers used in this study to amplify STR D06:54211118 are given in Supplementary Table S3.

## Results

### Genome-Wide Identification of Polymorphic STRs in *Gossypium hirsutum*

**Figure 1:**
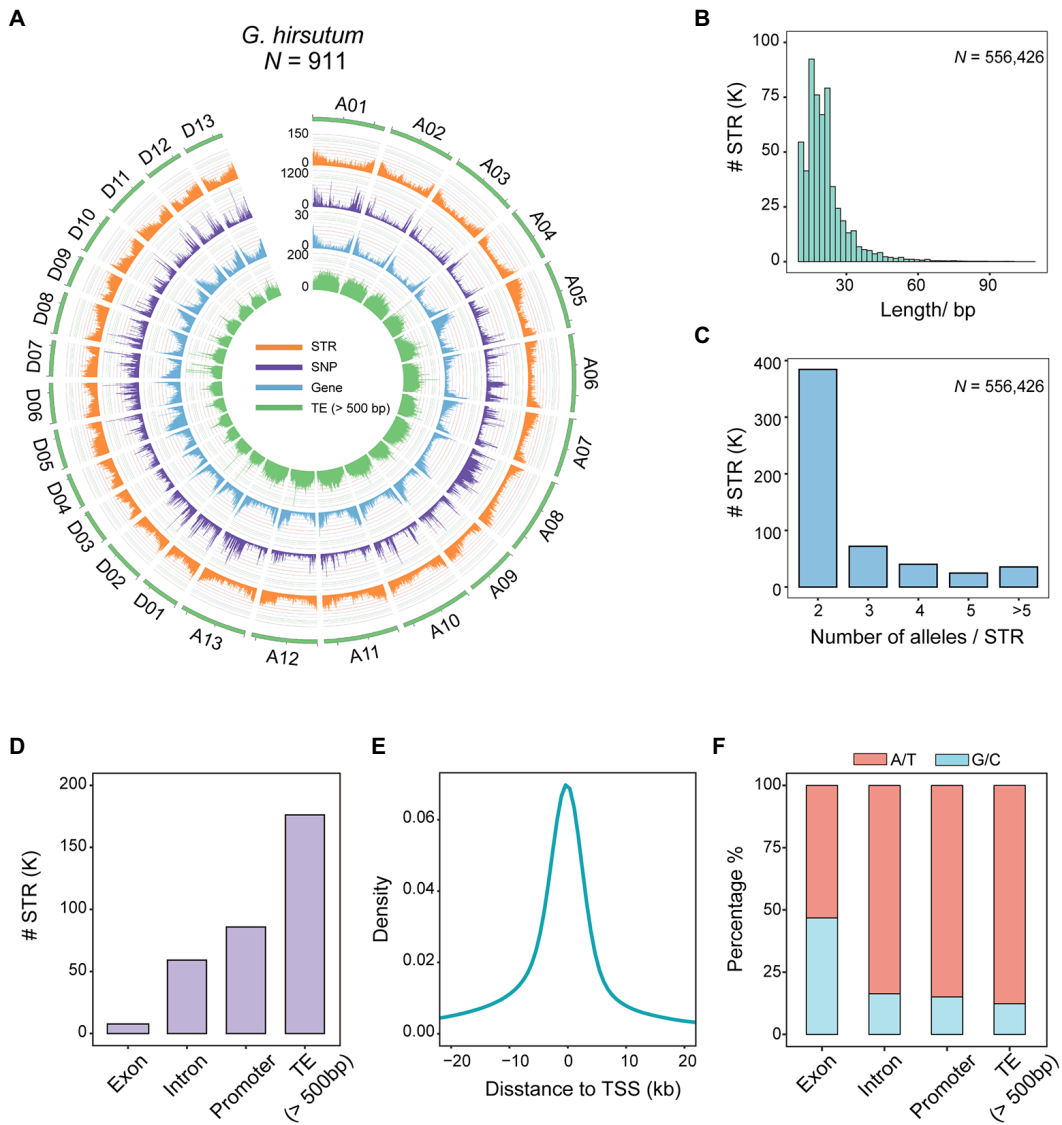
Polymorphic STR map of 911 cotton accessions. **(A)** Genomic distribution of polymorphic STRs in 911 allotetraploid cotton accessions. The 26 allotetraploid chromosomes, A01-D13, are represented in green. From outer ring to inner, curves represent the distributions of STRs, SNPs, genes, and TEs (>500 bp). **(B)** Distribution of STR motif lengths (length of the reference sequence at each locus). **(C)** Bar plot showing STR allele numbers. **(D)** Bar plot showing STR distributions within different genomic features [exons, introns, promoters, and TEs (>500 bp)]. **(E)** Density of STRs in relation to gene transcription start sites (TSSs). **(F)** CG composition of STR motifs according to associated genomic features.

To investigate STR polymorphisms in *G. hirsutum*, a global collection of 911 accessions from three independent GWAS panels was analyzed (Supplementary Table S4). These comprised 258 global modern improved cultivars or enhanced lines (termed P1; Fang et al., 2017), 234 commercial cultivars (termed P2; Fang et al., 2021), and 419 representative worldwide accessions released by Ma et al. (2018; termed P3). After quality control, reads of each accession were mapped to the reference genome *Gh* TM-1 V2.1 (Hu et al., 2019). Consecutively repeated units of 1–10 bp were identified from each aligned BAM file using HipSTR (Willems et al., 2017) in order to construct a polymorphic STR map. A total of 556,426 polymorphic STRs were identified (Figure 1A and Supplementary Table S5), which is far greater than the currently reported number of 100,290 STRs in the cotton genome (Wang et al., 2015). Among STRs identified here, 132,925 and 249,782 were shared by two and three datasets, respectively (Supplementary Figure S1). Interestingly, we found 70.5% (*n* = 392,116) of polymorphic STRs to also contain mutations such as SNPs and short indels (Supplementary Table S5).

Short tandem repeats were observed to be unevenly distributed across the genome, with a density of 0.25 per kb (Supplementary Table S6). Consistently, lower levels of STRs were observed in the centriole and terminals of each chromosome (Figure 1A), a finding in agreement with a previous report of greater SSR density in the distal gene-rich ends of chromosomes (Wang et al., 2015). Moreover, the genomic distribution of STRs was positively correlated with genes (*rho* = 0.89, *p* < 2.2 × 10^−16^, Spearman) and negatively correlated with transposable elements (TEs, >500 bp; *rho* = −0.81, *p* < 2.2 × 10^−16^, Spearman; Supplementary Figure S2). A higher density of STRs was observed on *GhDt* (*n* = 251,188, 0.31 per kb) than on *GhAt* (*n* = 305,238, 0.22 per kb; Supplementary Table S6). The motif length of STRs ranged from 11 to 108 bp, and 97.84% (544,400 out of 556,426) were shorter than 50 bp, with a mean of 21.1 bp, much shorter than the threshold for classification as structural variation (SV; >50 bp; Figure 1B and Supplementary Table S7). The majority of STRs (69.08%, *n* = 384,374) was biallelic, indicating a high level of polymorphism (Figure 1C and Supplementary Table S5).

In terms of genomic location, markedly fewer STRs were identified in exonic regions (1.39%, *n* = 7,718, termed exonic STRs) than in intronic regions (10.62%, *n* = 59,103) or transposons (31.67%, *n* = 176,245; Figure 1D). A total of 204,269 (36.71%) were sited close to genes, and particularly within 2,000 bp upstream of the transcriptional start sites (TSSs; Figure 1E and Supplementary Figure S3), indicating STRs as having potential to affect phenotypes by generating mutations in *cis* regulatory elements. We also examined the distribution of SNPs in relation to TSSs and found that less than 5% were located close to a TSS (<2 Kb; Supplementary Figures S3, S4). We further assessed STR motif types and the frequency with which different motifs occurred near a TSS (<2 Kb), and found that AT-rich motifs were more commonly enriched around TSSs (Supplementary Figure S5). In addition, the CG content of exonic STRs (46.74%, *n* = 88,732) was notably different from that in STRs of other regions (13.82%, *n* = 960,191), showing a significant CG bias in nucleotide composition (Figure 1F and Supplementary Table S8).

### Potential Effects of Polymorphic Exonic STRs on Genes

**Figure 2:**
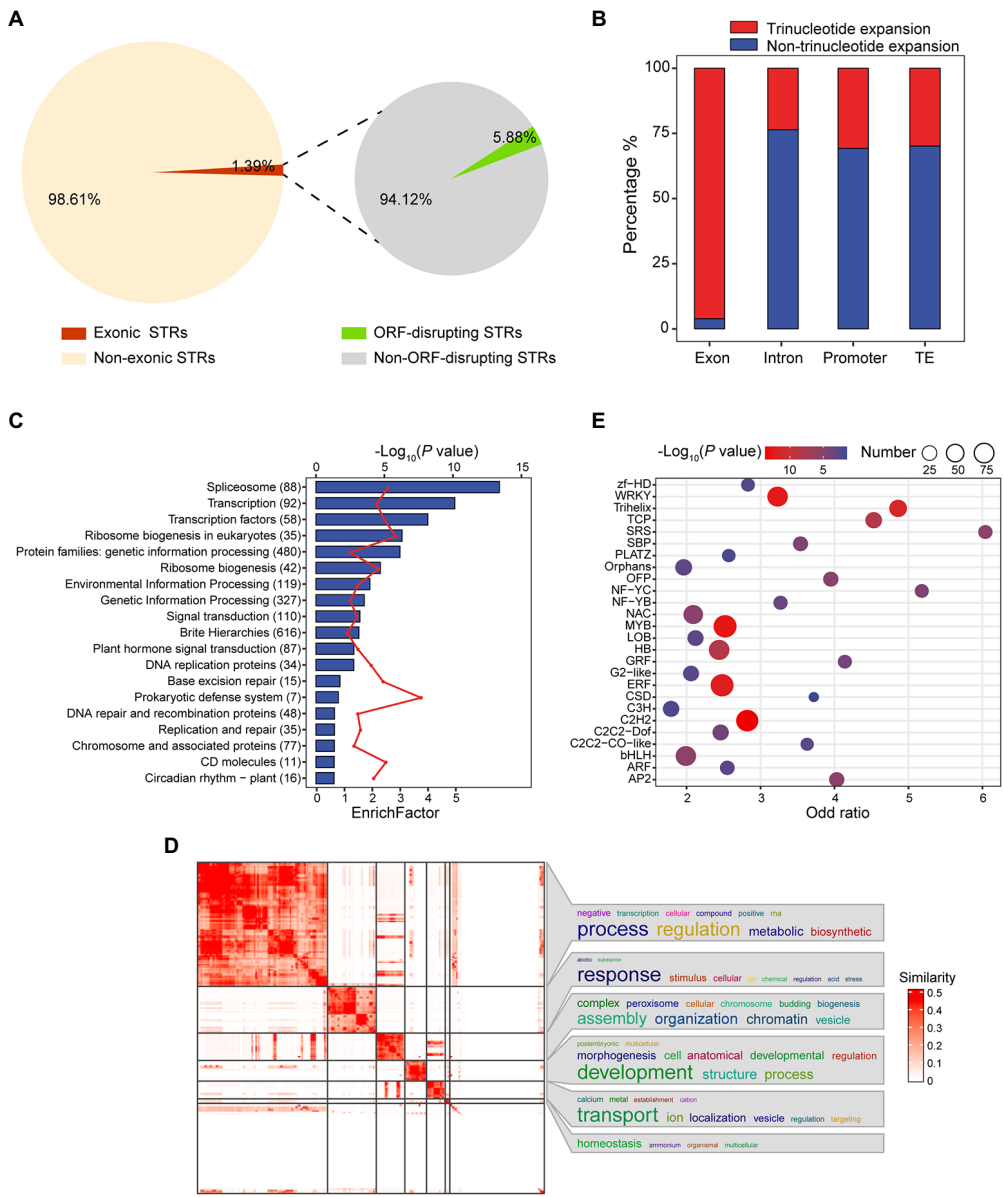
Investigating the impacts of STRs on genes. **(A)** Pie charts showing the proportion of exonic STRs and the distribution of exonic STRs causing frameshifts. **(B)** Proportion of length variation at each STR locus across different genomic annotation sets, where length variation was computed as |Ref(STR length) − Alt(STR length)|. **(C)** Bar diagram showing the KEGG analysis of 6,021 genes with exonic STRs. Blue bars represent −log_10_ (*p*), for which a greater value indicates higher enrichment degree. The red line represents the enrichFactor. The *y*-axis plots KEGG terms; numbers represent the genes included in each term. FDR < 0.01 was set as the cutoff. **(D)** Heat map showing similarities of significant GO biological process terms (FDR < 0.01) among the 6,021 genes with exonic STRs. The word cloud in the right panel visualizes the summarized biological functions in each GO cluster. GO enrichment analysis was performed using Fisher’s exact test and terms were clustered by the R package *simplifyEnrichment*. The color bar indicates similarity of GO terms. **(E)** Bubble chart of TF enrichment. Bubble size is directly proportional to the number of transcription factors. The *x*-axis represents odds ratio, while the *y*-axis lists transcription factors.

Exonic STRs have more potential to be important resources than do intergenic STRs. We identified 7,718 exonic STRs that were harbored by 6,021 genes (Supplementary Table S9), despite this category only accounting for 1.39% of total STRs (Figure 2A). Among these exonic STRs, only 5.88% altered the open reading frame (ORF) of the containing gene, including 26 transcription factors (TFs; Figure 2A and Supplementary Table S10). Interestingly, we found the overwhelming majority of exonic STRs (97.16%) to be trinucleotide, unlike those in introns (23.71%), promoters (30.57%), or TEs (29.26%; Figure 2B). We also found the expression levels of genes with non-trinucleotide exonic STRs were significantly lower compared to those with trinucleotide across multiple tissues (*p* < 0.05, student test; Supplementary Figure S6). In addition, it has been previously reported that STRs can have a *cis* regulatory effect on gene expression. We integrated both RNA-seq and whole-genome resequencing data to analyze the *cis* effects of STRs on gene expression. We identified 1,262 genes for which the presumed promoter contains a STR and determined that 8.95% of those genes (*n* = 113) exhibit different expression levels in association with different STR allels in promoter (Student’s *t*-test, *p* < 0.05; Supplementary Table S11).

Pathway enrichment analysis showed genes with exonic STRs to be significantly enriched in TFs (*p* = 7.29 × 10^−11^, Fisher’s exact test), ribosome biogenesis (*p* = 5.17 × 10^−7^, Fisher’s exact test), signal transduction (*p* = 6.10 × 10^−4^, Fisher’s exact test), and like terms (Figure 2C and Supplementary Table S12). GO analysis returned a similar result, with enrichment of terms such as regulation of transcription (GO:0006355, *p* = 2.16 × 10^−74^, Fisher’s exact test) and regulation of gene expression (GO:0010468, *p* = 5.35 × 10^−68^, Fisher’s exact test; Figure 2D, Supplementary Figure S7 and Supplementary Table S13). With respect to TFs, we found 974 genes coding for TFs that harbored exonic STRs. We tested each TF family for enrichment using Fisher’s exact test and revealed enrichment for exonic STRs in TFs related to stress, such as WRKY (*p* = 2.37 × 10^−13^, Fisher’s exact test, *n* = 62; Wang et al., 2021), ERF (*p* = 5.51 × 10^−13^, Fisher’s exact test, *n* = 93; Xie et al., 2019), and AP2 (*p* = 5.74 × 10^−6^, Fisher’s exact test, *n* = 18; Kazan, 2015), and also in those relating to fiber development, such as MYB (*p* = 1.26 × 10^−13^, Fisher’s exact test, *n* = 94; Wu et al., 2018) and NAC (*p* = 7.14 × 10^−6^, Fisher’s exact test, *n* = 52; Figure 2E and Supplementary Table S14).

Of genes containing exonic STRs, 572 are known to be involved in flowering (out of 4,562 flowering genes, Supplementary Table S2). Integrating this set with observed flowering traits (FD: days to flower; PP: plant period), we found at least 11 flowering genes for which STR genotypes corresponded to significant differences in traits (*p* < 0.05, Student’s *t*-test; Supplementary Table S15). For example, *GhTCP18* (*GH_D12G2879*) encodes the TF TCP18 and harbors a STR in its exon. In *Arabidopsis*, overexpression of *ATTCP18* (*AT3G18550*) in the shoot apical meristem leads to a late-flowering phenotype under both long-day and short-day conditions, mediated by interactions with the florigen proteins Flowing locus t (FT) and Twin sister of ft (TST; Aguilar-Martinez et al., 2007; Niwa et al., 2013).

Previous research reported a STR in *GhUBX* (*GH_D03G0985*) to have a significant correlation with fiber strength (FS; Supplementary Figure S8A), on account of influencing the interaction of GhUBX with GhSPL1 in the cortical microtubules of developing fibers (Zang et al., 2021). This variation of STR was also detected in P1 and P3 (Fang et al., 2017; Ma et al., 2018), with alleles of (GCCTCC)_5_ and (GCCTCC)_6_. The 6-bp variation segregated with the FS trait under different field conditions in multiple years (*p* = 8.40 × 10^−3^; Supplementary Figure S8B).

### Genome-Wide Association Analysis of STRs

**Figure 3:**
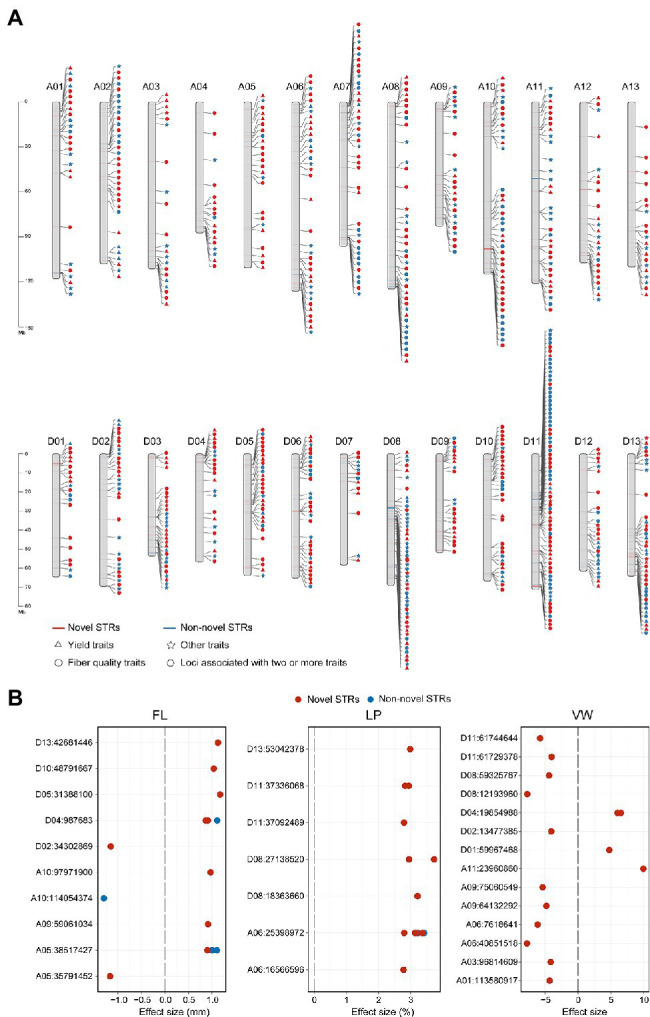
Distribution of 824 STR loci associated with agronomic traits. **(A)** Chromosomal distribution of 824 STR loci associated with agronomic traits. Red indicates novel STR loci, and blue those supported by previous SNP-based GWAS (non-novel STRs). Yield traits are BW, FWBP, LI, LP, SI, and BN (triangles). Fiber quality traits are FE, FL, FM, FS, LU, MAT, SCI, and FU (circles). Other traits are PH, VW, FD, and PP (stars). Hexagons represent STR loci associated with two or more traits (yield, quality, and other traits). A1-D13 represent the 26 allotetraploid cotton chromosomes. **(B)** Effect sizes of STRs associated with FL, LP, and VW. Red circles represent novel STR loci and blue circles non-novel STRs. Each dot indicates an environment.

To address whether STRs could contribute to agronomic traits, we performed a STR-GWAS in P1, P2, and P3 cotton accessions originally subjected to SNP-based GWAS (Fang et al., 2017, 2021; Ma et al., 2018) in relation to 18 traits (Supplementary Figure S9). After strict quality control, we, respectively, obtained 14,241 (Fang et al., 2017), 8,504 (Fang et al., 2021), and 36,557 (Ma et al., 2018) high-quality biallelic STRs (MAF > 0.05, missing ratio <30%) from P1, P2, and P3. LD analysis indicated that the scope of LD among STRs ranged from 300 to 400 kb, showed a low LD with the surrounding SNPs (Supplementary Figure S10; Fang et al., 2017), and their MAF ranged from 0.185 to 0.197 (Supplementary Figure S11). We then identified trait-associated STRs using Efficient Mixed-Model Association eXpedited (Emmax; Kang et al., 2010), which yielded a total of 824 significant STRs including 214 associated with yield, 422 associated with fiber quality, and 228 associated with other traits (Figure 3A and Supplementary Table S16). Of the GWAS loci, 333 (40.41%) were supported by previous SNP-based GWAS due to the associated STR and SNP falling in the same LD block. Next, we examined the functional effects of these STRs in relation to specific traits and classified them into 18 groups based on their functional consequences (Figure 3B and Supplementary Figure S12). For instance, one group comprised STRs affecting lint percentage (LP) and included STR A06:25398972, the alternate allele of which increased LP by 3% in multiple environments.

**Figure 4:**
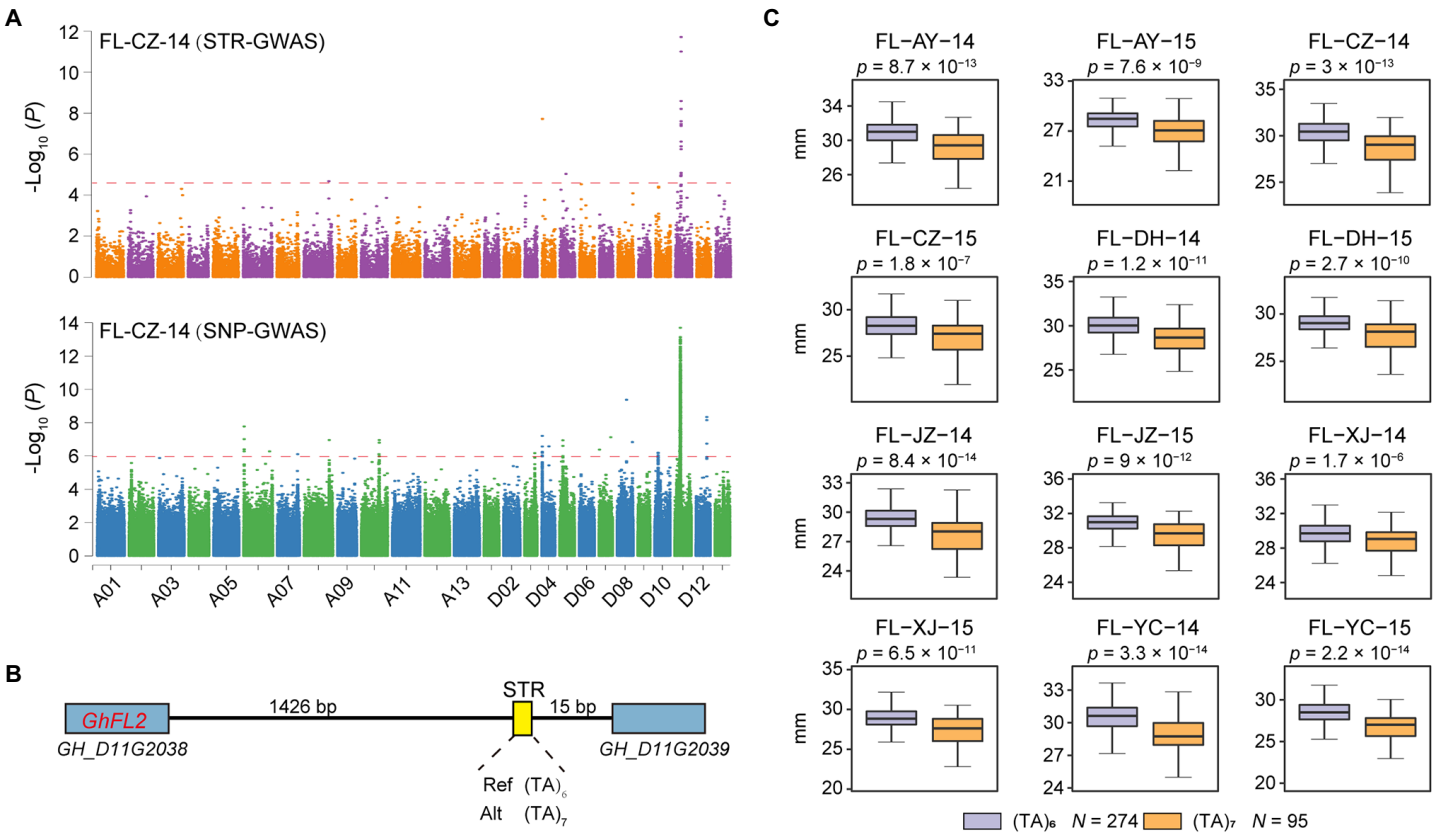
The STR D11:23844508 associated with fiber length. **(A)** Combined Manhattan plot for FL based on STRs (top) and SNPs (bottom) in P3. **(B)** The LocusZoom plot (D11 from 23.843 to 23.845 Mb). **(C)** Box plot for FL according to the two haplotypes of STR D11:23844508 in P3 (*n* = 274 vs. 95). Center line, median; box limits, upper and lower quartiles; and whiskers, 1.5× the interquartile range (^*^p < 0.05, two-sided *t*-test). AY: Anyang in Henan Province; CZ: Cangzhou in Hebei Province; DH: Dunhuang in Gansu Province; JZ: Jingzhou in Hubei Province; XJ: Alaer in the Xinjiang; and YC: Yancheng in Jiangsu Province. 14: 2014; 15: 2015.

As another example, a locus associated with fiber length on chromosome D11 was previously detected by SNP-based GWAS (Figure 4A). The SNP-based study reported and validated *GhFL2* (*GH_D11G2038*) as a candidate gene controlling fiber development (Ma et al., 2018), but STR variation of this locus was ignored. We identified a lead STR (D11:23844508) located 1.4 Kb downstream of *GhFL2* (Figure 4B) for which the (TA)_6_ allele was associated with 3.69–6.44% greater fiber length (FL) compared to the (TA)_7_ allele (*p* < 1.7 × 10^−6^, Student’s *t*-test; Figure 4C).

In addition to direct fiber traits, early maturation of cotton cultivars is important for cotton breeding. We obtained 47 STRs associated with days to flower (FD), of which 66% (*n* = 31) were located on A03. We also detected a STR on D03 (D03:32951122; Supplementary Figure S13A), at the 85 Kb upstream of *GH_D03G0916* (*GhUCE*; Supplementary Figure S13B), which encodes an ubiquitin-conjugating enzyme. Overexpression of *GhUCE* results in early flowering and fewer rosette leaves relative to wild type (Ma et al., 2018). In the present study, accessions with the (TATCTG)_6_ allele (*n* = 392) had FD reduced by 2.34–14.21 days relative to those with the (TATCTG)_4_ allele (*n* = 21; *p* < 4.9×10^−4^, Student’s *t*-test) in seven out of nine examined environments (Supplementary Figure S13C). These results support that STRs could be utilized as molecular breeding markers.

### Novel STR Loci Associated With Agronomic Traits

**Figure 5:**
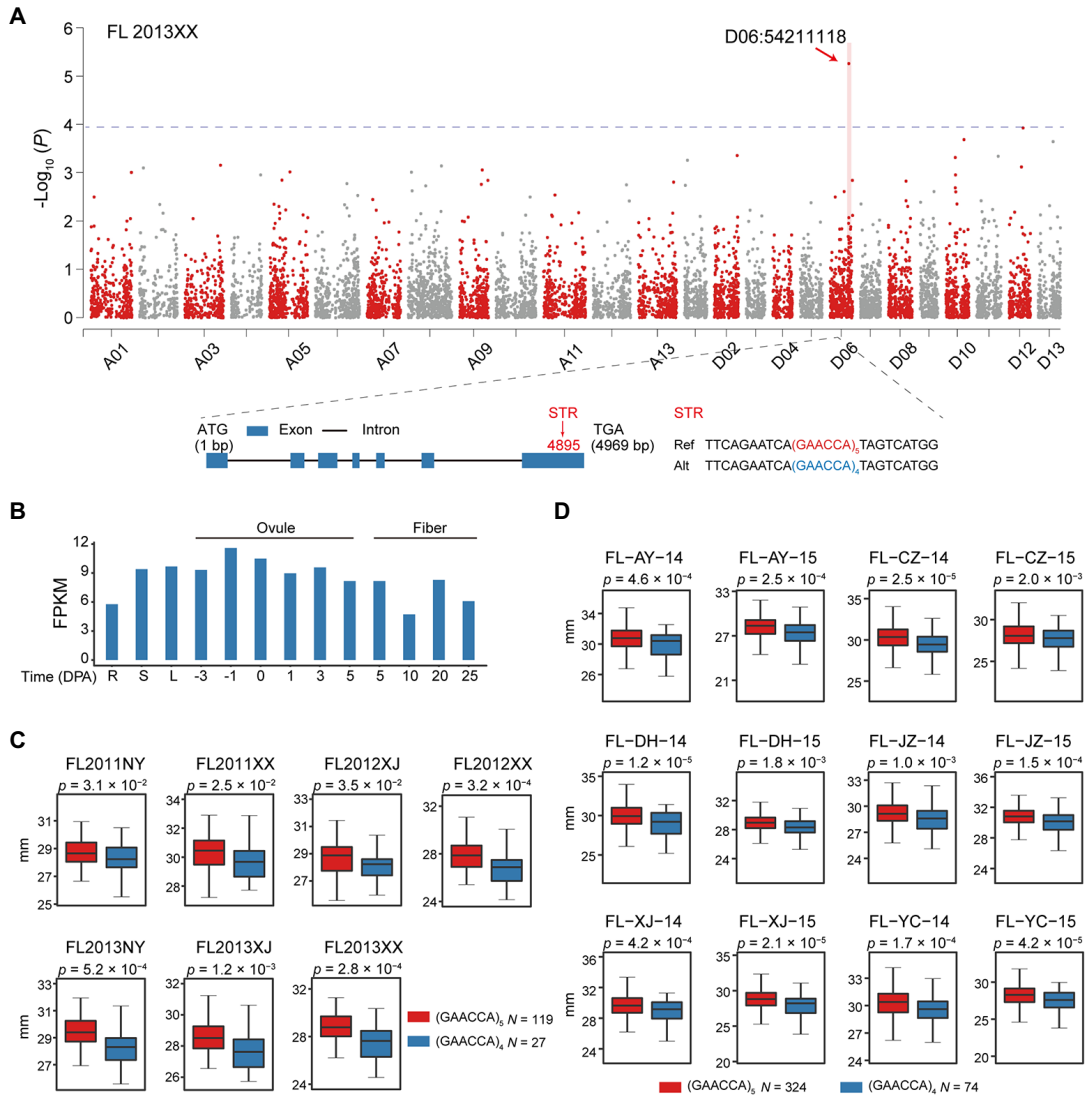
Identification of a candidate gene, *GH_D06G1697*, harboring a polymorphic STR (D06:54211118) associated with fiber length. **(A)** Manhattan plot for fiber length. Statistical analysis was performed with the two-sided *t*-test in P2. Exon-intron structure of *GH_D06G1697* and the exonic STR D06:54211118. Blue rectangles and black lines, respectively, indicate exons and introns. Ref, reference; Alt, alternate. **(B)** Expression level of *GH_D06G1697* during ovule and fiber development stages in different tissues, including root (R), stem (S), and leaf (L), with values as FPKM. **(C)** Box plot of fiber length in relation to genotype of the exonic STR D06:54211118 (*n* = 119 vs. 27) in P2. Center line, median; box limits, upper and lower quartiles; and whiskers, 1.5× the interquartile range (two-sided *t*-test). NY: Nanyang in Henan province; XX: Xinxiang in Henan province; and XJ: Korla in Xinjiang. **(D)** Box plot of fiber length in relation to STR genotype (*n* = 324 vs. 74) in P3. Center line, median; box limits, upper and lower quartiles; and whiskers, 1.5× the interquartile range (two-sided *t*-test). AY: Anyang in Henan Province; CZ: Cangzhou in Hebei Province; DH: Dunhuang in Gansu Province; JZ: Jingzhou in Hubei Province; XJ: Alaer in the Xinjiang; and YC: Yancheng in Jiangsu Province. 14: 2014; 15: 2015.

Overall, we identified 491 STRs that had not previously been detected by GWAS (Supplementary Table S16). One notable example is a STR D06:54211118 associated with FL in seven environments; this variation is located in an exon of *GH_D06G1697*, which encodes Sn1-specific diacylglycerol lipase alpha (Figure 5A) and is characterized by a (GAACCA)*_n_* repeat that can cause deletion of a glutamine and asparagine (Figure 5A). Expression analysis of *GH_D06G1697* showed the gene to be consistently expressed in most tissues, including ovules and fibers at different developmental stages (Figure 5B). Expression analysis in 65 individuals showed that the reference (GAACCA)_5_ allele does alter expression of *GH_D06G1697* (*p* < 1.4 × 10^−5^, Student’s *t*-test; Supplementary Figure S14 and Supplementary Table S17). Plants with that allele (*n* = 119) exhibited significantly increased FL (1.96–4.83% greater, *p* < 3.5 × 10^−2^, Student’s *t*-test) over those having the alternate (GAACCA)_4_ allele (*n* = 27; Figure 5C).

To further examine whether the effect of STR D06:54211118 on FL is common across upland cotton, we utilized a previously published GWAS population (Ma et al., 2018). In 12 environments, *GH_D06G1697* with the (GAACCA)_5_ allele (reference, *n* = 324) resulted in significantly increased FL (1.84–3.38% greater, *p* < 1.0 × 10^−3^, Student’s *t*-test) over the (GAACCA)_4_ allele (alternate, *n* = 74) in P2 (Figure 5D). As the STR D06:54211118 was not supported by prior GWAS, we selected 16 accessions according to genotype [(GAACCA)_5_, *n* = 8; (GAACCA)_4_, *n* = 8; Supplementary Figure S15] and verified the locus by PCR (Supplementary Figure S16 and Supplementary Table S3). This result supports that *GH_D06G1697* may be a novel gene controlling fiber development.

We furthermore compared the sequence of this STR in allotetraploid (AADD) *Gb* and *Gh* and the two diploid cotton species presumed their ancestors, *G. arboretum* (*Ga*; AA) and *G. raimondii* (*Gr*; DD; Supplementary Figure S17). We identified a genotype of (GAACCA)_3_ in *Ga* and the A subgenomes from *Gb* and *Gh*, but a range of 2–5 repeats in *Gr* and the D subgenomes from *Gb* and *Gh* (Supplementary Figure S18 and Supplementary Table S3). This indicates the STR variation of *GH_D06G1697* to have occurred only in the D subgenome during the polyploidization and differentiation of *Gh* and *Gb*.

### Utility of a Web-Based Application for STR Datasets

Trait-associated STRs would constitute a considerable resource for better understanding the genetic basis of traits and also provide new markers for marker-assisted breeding of *G. hirsutum*. Here, we developed a database named CottonSTRDB.^5^ It contains a wealth of information on the 556,426 STRs identified in this study, including their chromosome locations, putative functions, polymorphism among genotypes, and the results of our STR-GWAS (Supplementary Figure S19). The markers can be searched using multiple parameters including chromosome number(s), chromosome/scaffold location, motif type, associated loci, and genotypes of STR loci, which can be downloaded in TXT format. All results can be downloaded in CSV format. If a breeder is interested in a specific trait, information can be retrieved on STRs involved in that trait. Thus, our database will hasten the process of developing candidate gene markers.

## Discussion

In crop, the identification of STRs typically relied on the presence of STR motifs in DNA sequences (Wang et al., 2015; Wu et al., 2020) and the variation found in one individual were limited (Wang et al., 2015). To date, few population-scale studies identifying STRs have been reported in crops. In this study, we constructed a genome-wide and population-scale polymorphic STR map of *G. hirsutum* with 556,426 STRs that integrated 911 cotton genomes (Fang et al., 2017, 2021; Ma et al., 2018). We performed an integrative study involving detailed characterization of STR in the cotton genome and their impact on gene structure and phenotype. Moreover, we constructed a platform, named CottonSTRDB, based on collective data from the abundant publications on cotton genetics and GWAS cohorts. This database includes genotype information for STRs related to traits of interest, along with their effect sizes. Our results provide insights into functional roles of STRs in influencing complex traits and will serve the cotton community as a valuable resource for molecular breeding.

Short tandem repeats were previously considered as evolutionarily neutral DNA sequence (Awadalla and Ritland, 1997). Recent study and our analysis demonstrated that STR distribution is nonrandom: (i) STR distribution and effect in gene expression. Distinct from SNPs, STRs in the cotton genome have higher extent near TSSs and within coding exons compared to other regions (Supplementary Figures S3, S4). We found 8.95% of those genes (*n* = 113) exhibited different expression levels in association with different STR alleles at promotor (Student’s *t*-test, *p* < 0.05). This result suggests that the STR can work as cis-acting sequences to regulate the levels of expression. (ii) Evolutionary significance in different species. Genes with STRs have biological relevance in diverse species. In cotton, 8.26% of genes harbored exonic STRs, similar to previously reported values ranging from 13.1 to 21% in *Caenorhabditis elegans*, *A. thaliana*, and *Drosophila melanogaster* (Gemayel et al., 2010). The genes with exonic STR have mostly identical function descriptions, which were implicated in processes such as regulation of transcription, consistent with previous studies in yeast (Richard and Dujon, 2006) and humans (Legendre et al., 2007).

We employed GWAS to determine the likelihood that each STR causally affects trait. We identified a total of 824 significant STRs related to specific traits. Interestingly, 491 novel STR-GWAS signals that cannot be detected by regular SNP-GWAS. The phenomenon can be explained by different mutation ratios between SNP and STR. Indeed, most STR showed a low LD with the surrounding SNPs; thus, some of the STRs were not being in high linkage disequilibrium with any SNP. Our result showed STR can also reveal additional association loci. Due to the limited mapping resolution of GWAS, most our STR-GWAS findings are associative, not causative. Moreover, various molecular mechanisms have been proposed of STR (Fotsing et al., 2019). Analysis using STR combined with transcriptomic, proteomic and metabonomics can help to study STR function. Recently, two population-scale transcriptomes have been released in cotton community (Li et al., 2020; Ma et al., 2021a). Further work should integrate genomic and transcriptomic statistical analysis of eSTR to identify causative trait-associated STR.

Additionally, STR has a high mutation rate related to SNP and exhibits evolutionary significance. The change in STR may provide immediate benefits in adaptation to varying ecological factors, such as biotic, environmental, and climate change. For example, we found 47 STR associated with days to flower. We detected a STR on D03 (D03:32951122; Supplementary Figure S13), accessions with the (TATCTG)_6_ allele (*n* = 392) had FD reduced by 2.34–14.21 days relative to those with the (TATCTG)_4_ allele (*n* = 21; *p* < 4.9 × 10^−4^, Student’s *t*-test) in seven out of nine examined environments. It is therefore to intriguing to explore the STR associated with environmental factor.

## Conclusion

The study was designed to determine whether STR could provide a strategy for allele mining in crop. A total of 911 individuals from three independent GWAS cohorts was genotyped for the STR variations. A total of 556,426 polymorphic STRs were well categorized, including their genomic location, linkage disequilibrium, impact on gene structure, and their potentially association with specific traits. We found that 824 STRs were significantly associated with agronomic traits, including 491 novel alleles. The database CottonSTRDB was further developed to facilitate use of STR datasets for complex traits. Our study provides an alternative strategy STR-GWAS for allele mining and valuable resource for cotton breeding.

## Data Availability Statement

The datasets presented in this study can be found in online repositories. The names of the repository/repositories and accession number(s) can be found in the article/**Supplementary Material**.

## Author Contributions

LF and TnZ conceptualized the project. HM, TnZ, ZD, and YH performed the bioinformatics analysis. HM, JH, BX, RC, JunZ, and JuncZ extracted high-quality DNA and performed PCRs for STR genotyping. LF, TnZ, HM, and TaZ prepared the manuscript. All authors contributed to the article and approved the submitted version.

## Funding

This study was financially supported in part by grants from projects of the Hainan Yazhou Bay Seed Lab (B21HJ0223), the NSFC (32172008 and 31822036), the Leading Innovative and Entrepreneur Team Introduction Program of Zhejiang (2019R01002), a project from Sanya Yazhouwan Technology City (SKJC-2021-02-001), and the Fundamental Research Funds for the Central Universities (2020XZZX004-03).

## Conflict of Interest

The authors declare that the research was conducted in the absence of any commercial or financial relationships that could be construed as a potential conflict of interest.

## Publisher’s Note

All claims expressed in this article are solely those of the authors and do not necessarily represent those of their affiliated organizations, or those of the publisher, the editors and the reviewers. Any product that may be evaluated in this article, or claim that may be made by its manufacturer, is not guaranteed or endorsed by the publisher.

## Footnotes

^1^ http://cotton.zju.edu.cn/

^2^ http://broadinstitute.github.io/picard/

^3^ http://pgrc.ipk-gatersleben.de/misa/misa.html

^4^ https://bioconductor.org/packages/simplifyEnrichment/

^5^ https://www.datanewlook.com/shiny/str/
