# Supplementary material for: Population-Scale Polymorphic Short Tandem Repeat Provides an Alternative Strategy for Allele Mining in Cotton
Source: Front Plant Sci. 2022 May 6;13:916830. doi: 10.3389/fpls.2022.916830 (PMC9120961; doi:10.3389/fpls.2022.916830)
Supplement: Supplementary file 1 [file Data_Sheet_1.docx]

**Population-scale Polymorphic Short Tandem Repeat Provide an Alternative Strategy for Allele Mining in Cotton**

Huan Mei^1§^, Ting Zhao^1§^, Zeyu Dong^1^, Jin Han^1^, Biyu Xu^1^, Rui Chen^1^, Jun Zhang^1^, Juncheng Zhang^1^, Yan Hu^1,2^, Tianzhen Zhang^1,2^, Lei Fang^1,2*^

^1^ Zhejiang Provincial Key Laboratory of Crop Genetic Resources, Institute of Crop Science, Plant Precision Breeding Academy, College of Agriculture and Biotechnology, Zhejiang University, Hangzhou, China

^2^ Hainan Institute of Zhejiang University, Sanya, China

^§^ These authors contributed equally to this work.

*Correspondence and requests for materials should be addressed to Lei Fang ([fangl@zju.edu.cn)](mailto:xueyingguan@zju.edu.cn)).

**Supplementary Figure S1 - S19**

**Figure S1.** Venn diagram showing the overlapping of STRs in P1, P2 and P3 population.

**Figure S2.** Genome-wide distributions of STRs, genes, and TEs (> 500 bp) in the assembled genome of *G. hirsutum*.

**Figure S3.** The percentage of 556,426 polymorphic STRs in relation to TSS.

**Figure S4.** The Distance distribution of SNP to closely genes.

**Figure S5.** STR motif type and frequency of the STR motif in relation to TSS (< 2 Kb).

**Figure S6.** Box plot for expression of genes with trinucleotide STRs and genes with non-trinucleotide STRs.

**Figure S7.** GO enrichment of 6,021 genes with exonic STRs.

**Figure S8.** *GhUBX* with polymorphic STR D03:34970506 associated with fiber strength.

**Figure S9.** Bar graph for the analogy and group source of the traits used for STR-GWAS.

**Figure S10.** LD decay estimated from populations P1, P2, P3.

**Figure S11.** Density of MAF for STRs in three populations.

**Figure S12.** Effect size of STRs associated with agronomic traits.

**Figure S13.** Association of STR D03:32951122 with days to flower (FD) in P3.

**Figure S14.** Box plot for expression of *GH_D06G1697* based on the two haplotypes of D06:54211118 STR.

**Figure S15**. Polymorphism of STR D06:54211118 for 16 accessions in P1 based on resequencing data.

**Figure S16.** PCR based verification for the polymorphism of STR D06:54211118 for 16 accessions in P1 population.

**Figure S17.** Genotyping of STR D06:54211118 in *Gossypium* species.

**Figure S18.** PCR based verification of STR D06:54211118 in *Gossypium* species.

**Figure S19.** The user-friendly interface of CottonSTRDB.

**Supplementary Table S1-S17**

**Table S1**. Phenotypic data used for STR-GWAS. (**Separate files**)

**Table S2**. List of 4,562 flowering genes in *Gossypium hirsutum*. (**Separate files**)

**Table S3**. Primers sequence for PCR of STR D06_54211118 in this study.

**Table S4.** Summary of 911 cotton accessions used in this study. (**Separate files**)

**Table S5.** Genome wide identification of 556,426 polymorphic STRs in *G. hirsutum.* (**Separate files**)

**Table S6**. Summary of chromosomal distribution and average density of STRs mapped on *G. hirsutum* chromosomes.

**Table S7**. The distribution of allele length across all genotyped polymorphic STRs. (**Separate files**)

**Table S8**. Base-composition pattern of polymorphic STRs across different genomic features. (**Separate files**)

**Table S9.** A list of 6,021 genes with exonic STRs. (**Separate files**)

**Table S10**. List of exonic STRs changing the open read frame (ORF) of genes. (**Separate files**)

**Table S11.** Promotor STRs associated with the expression of the genes. (**Separate files**)

**Table S12**. Pathway enrichment result of 6,021 genes with exonic STRs. (**Separate files**)

**Table S13**. Gene Ontology (GO) enrichment of biological processes linked to 6,021 genes with exonic STRs. (**Separate files**)

**Table S14.** Enriched transcription factors with exonic STRs in 6,021 genes.

**Table S15**. Flowering genes exhibiting significantly difference according to the genotype of STRs.

**Table S16**. Significant association loci of STR-GWAS. (**Separate files**)

**Table S17** Expression of *GH_D06G1697* based on the two haplotypes of D06:54211118 STR.

**Figure S1.** **Venn diagram showing the overlapping of STRs in P1, P2 and P3 population.**


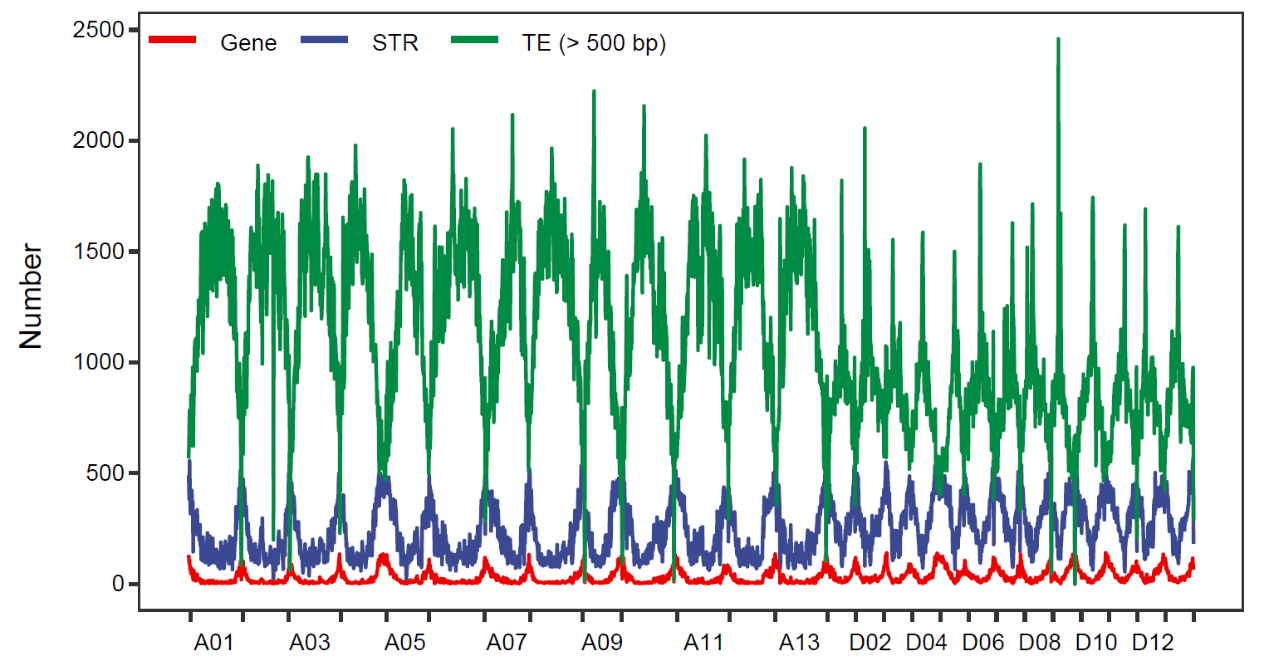


**Figure S2. Genome-wide distributions of STRs, genes, and TEs (> 500 bp) in the assembled genome of *G. hirsutum***.

The horizontal axis indicates the chromosomes A01-A13 and D01-D13, and each chromosome was divided into 1-Mb for statistical analysis. The left vertical axis indicates the frequencies of STRs, genes, and TEs (> 500 bp). The curves represent practical frequencies.


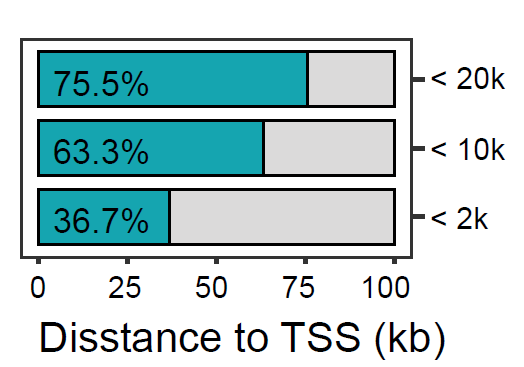


**Figure S3. The percentage of 556,426 polymorphic STRs in relation to TSS.**

The distance to TSS (transcriptional start site) was divided into < 2 Kb, < 10 Kb and < 20 Kb.


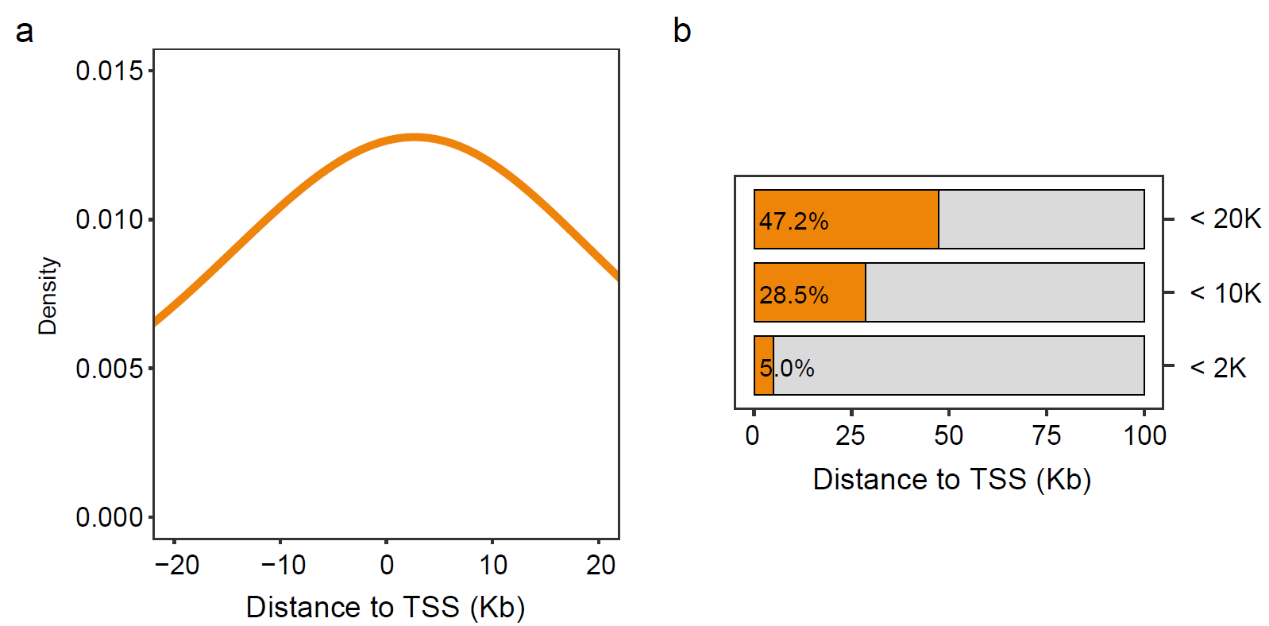


**Figure S4. The Distance distribution of SNP to closely genes.**

(a) Density of SNPs in relation to closely gene TSS. (b) Percentage of SNPs in relation to TSS. The distance was divided into < 2 Kb, < 10 Kb and < 20 Kb.

**
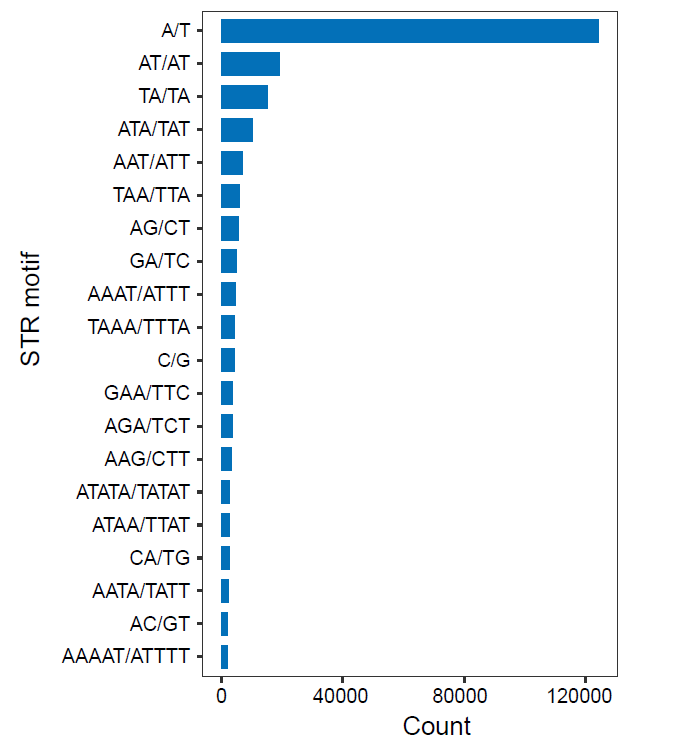
**

**Figure S5. STR motif type and frequency of the STR** **motif in relation to TSS (< 2 Kb).**


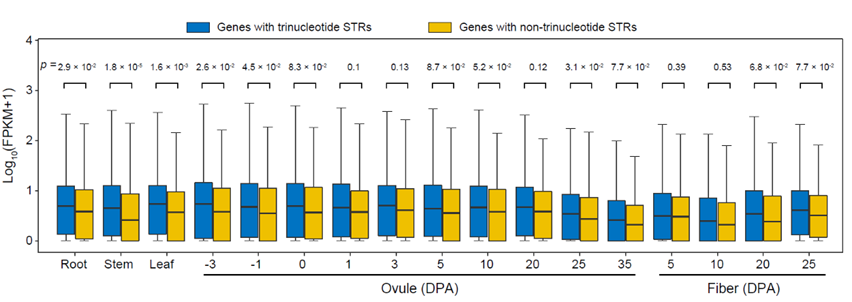


**Figure S6. Box plot for expression of genes with trinucleotide STRs and genes with non-trinucleotide STRs.**

Center line, median; box limits, upper and lower quartiles; whiskers, 1.5× the interquartile range.


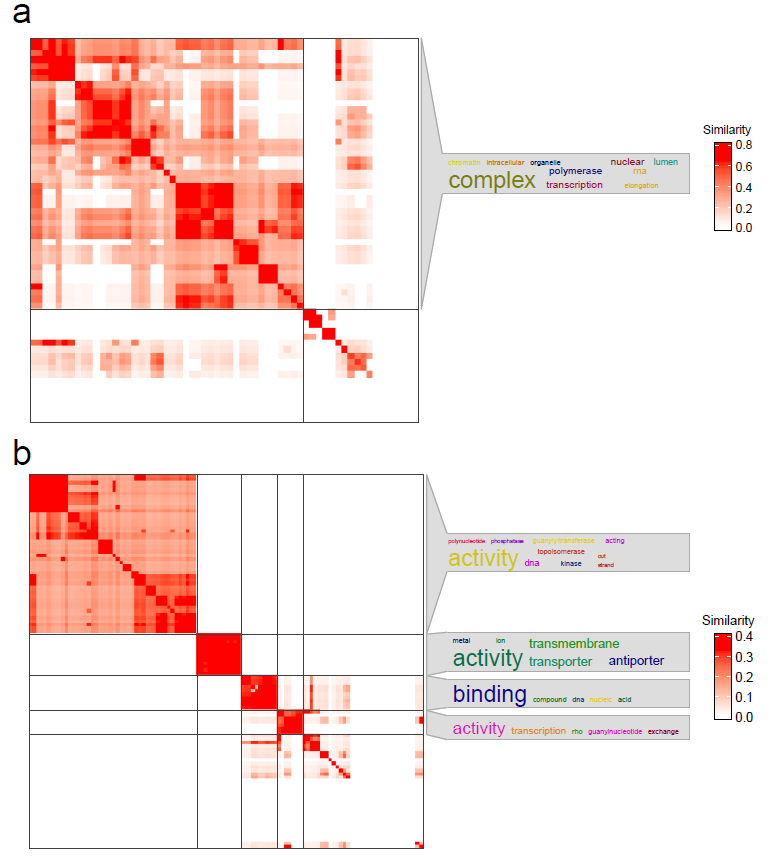


**Figure S7. GO enrichment of 6,021 genes with exonic STRs.**

Heatmap showing similarities of significant GO cellular component terms (a) and molecular function (b) (FDR < 0.01) among the 6,021 genes with exonic STRs. The word in the right panel visualizes the summarized biological functions in each GO cluster. The color bar indicates similarity of GO terms.


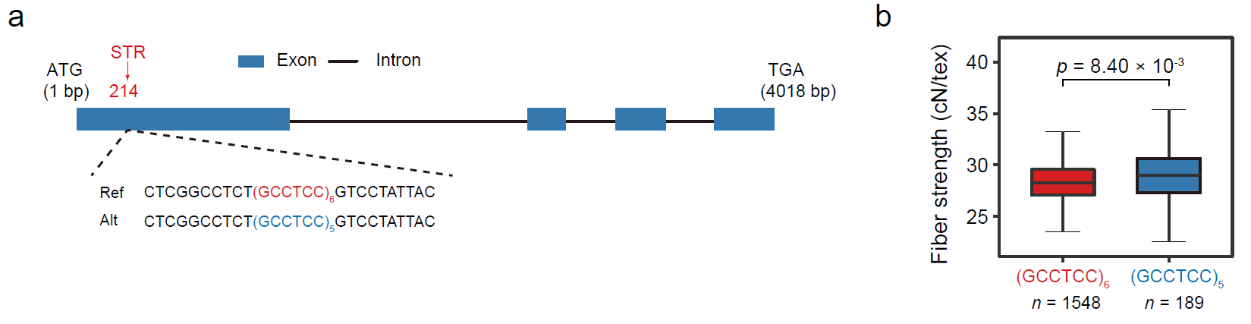


**Figure S8. *GhUBX* with polymorphic STR D03:34970506 associated with fiber strength (FS).**

**(a)** Exon–intron structure of *GhUBX* and the exonic STR D03:34970506. Blue rectangles and black line respectively indicate exons and introns. Ref, reference; Alt, alternate. **(b)** Genotype association analysis of the STR D03:34970506 for FS (*n* = 1548 vs 189) in P1. Center line, median; box limits, upper and lower quartiles; whiskers, 1.5× the interquartile range (**p* < 0.05, Student’s t-test).


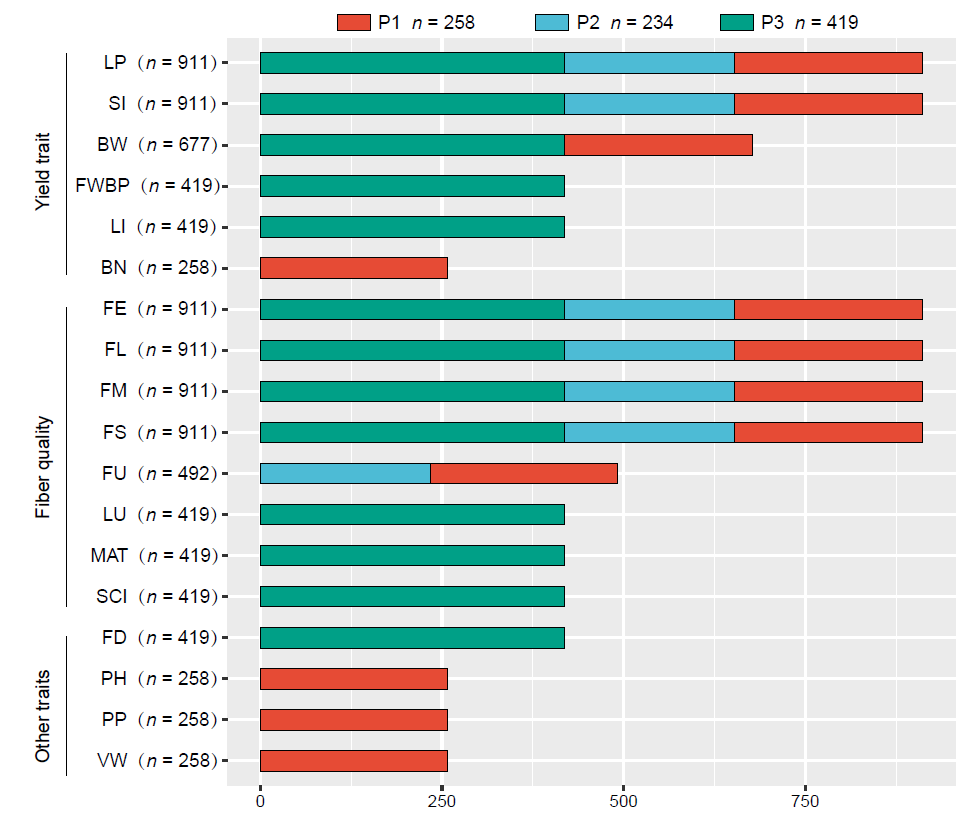


**Figure S9. Bar graph for the analogy and group source of the traits used for STR-GWAS.**

LP, lint percentage; SI, seed index; BW, Boll weight; FWBP, Fiber weight per boll; LI Lint index; BN, Boll number; FE, Fiber elongation; FL, Fiber length; FM, Fiber micronaire; FS, Fiber strength; FU, Fiber uniformity; LU, Length uniformity; MAT, Maturity; SCI, Spinning consistency index; FD, Days to flower; PH, Plant height; PP, Plant period; VW, Verticillium wilt.


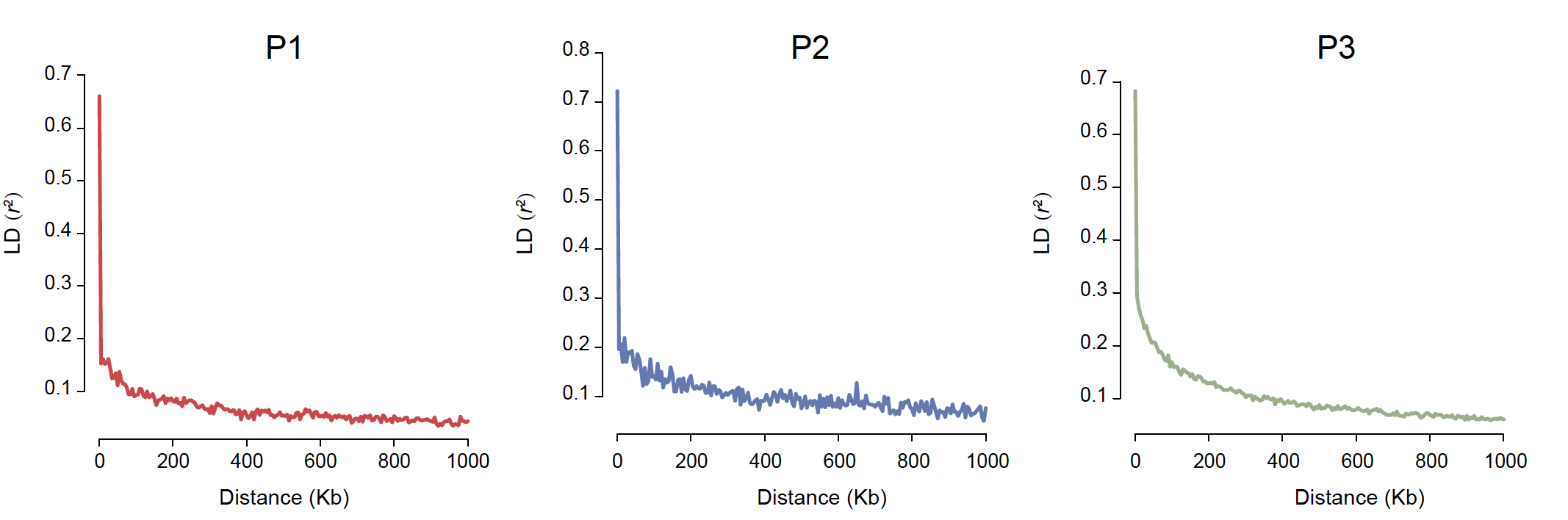


**Figure S10.** **LD decay estimated in populations P1, P2, P3.** LD decay determined by squared correlations of allele frequencies (*r*^2^) against distance between polymorphic sites.

**
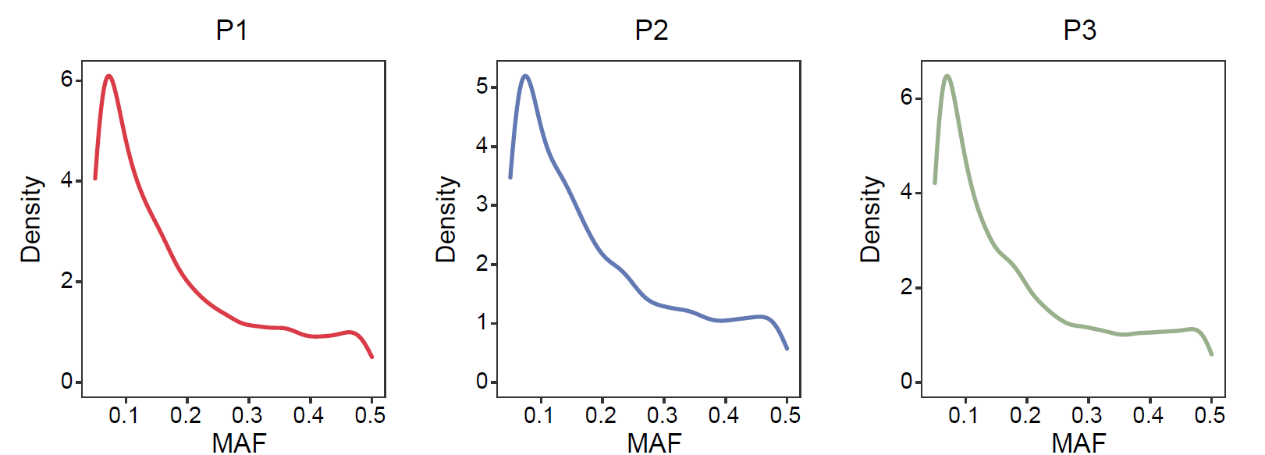
**

**Figure S11. Density of MAF for STRs in three populations.**


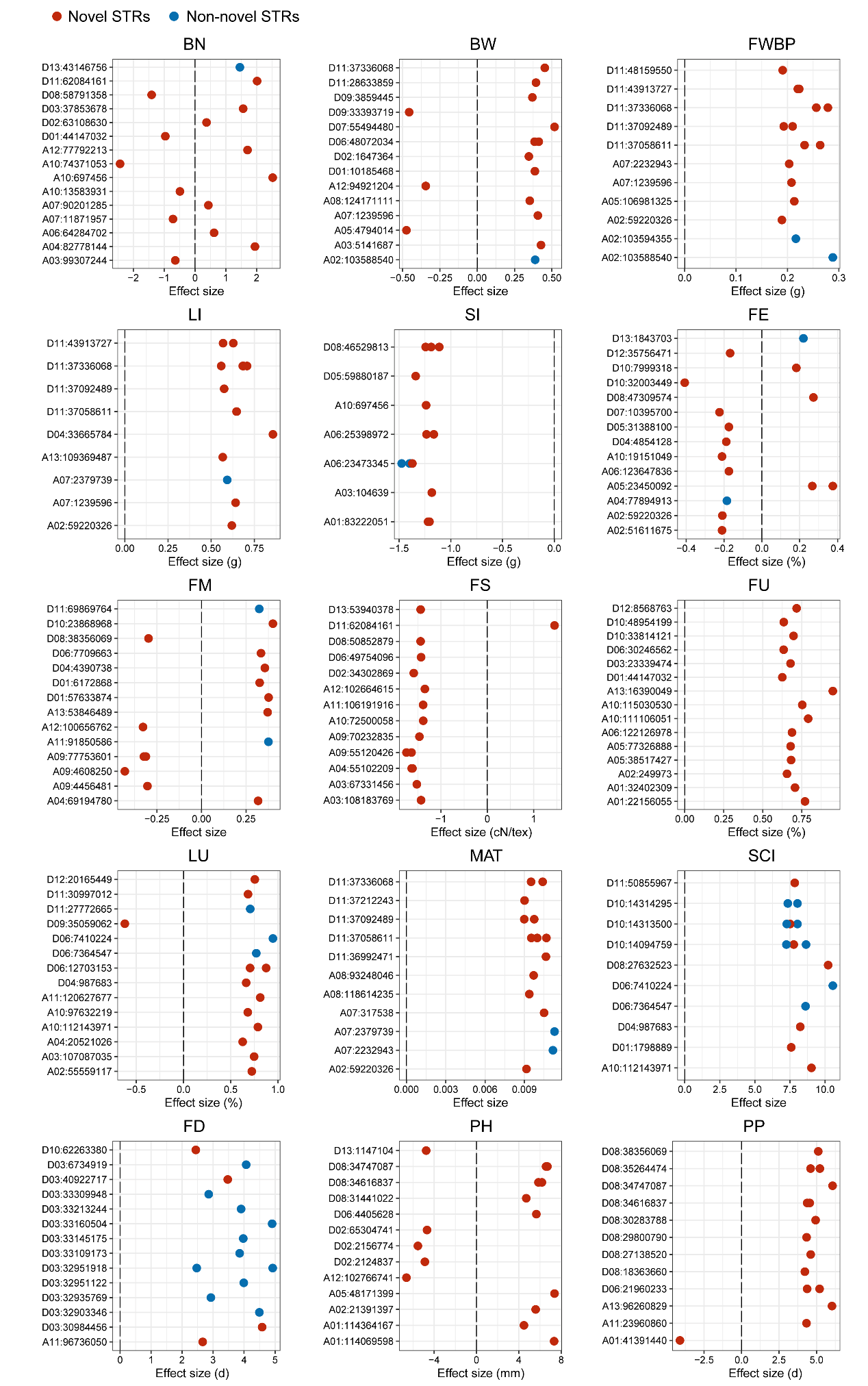


**Figure S12. Effect size of STRs associated with agronomic traits.**

Agronomic traits included BN, BW, FWBP, LI, SI, FE, FM, FS, FU, LU, MAT, SCI, FD, PH, PP. Red circle represents the novel STR loci and blue circle represents non-novel STRs.


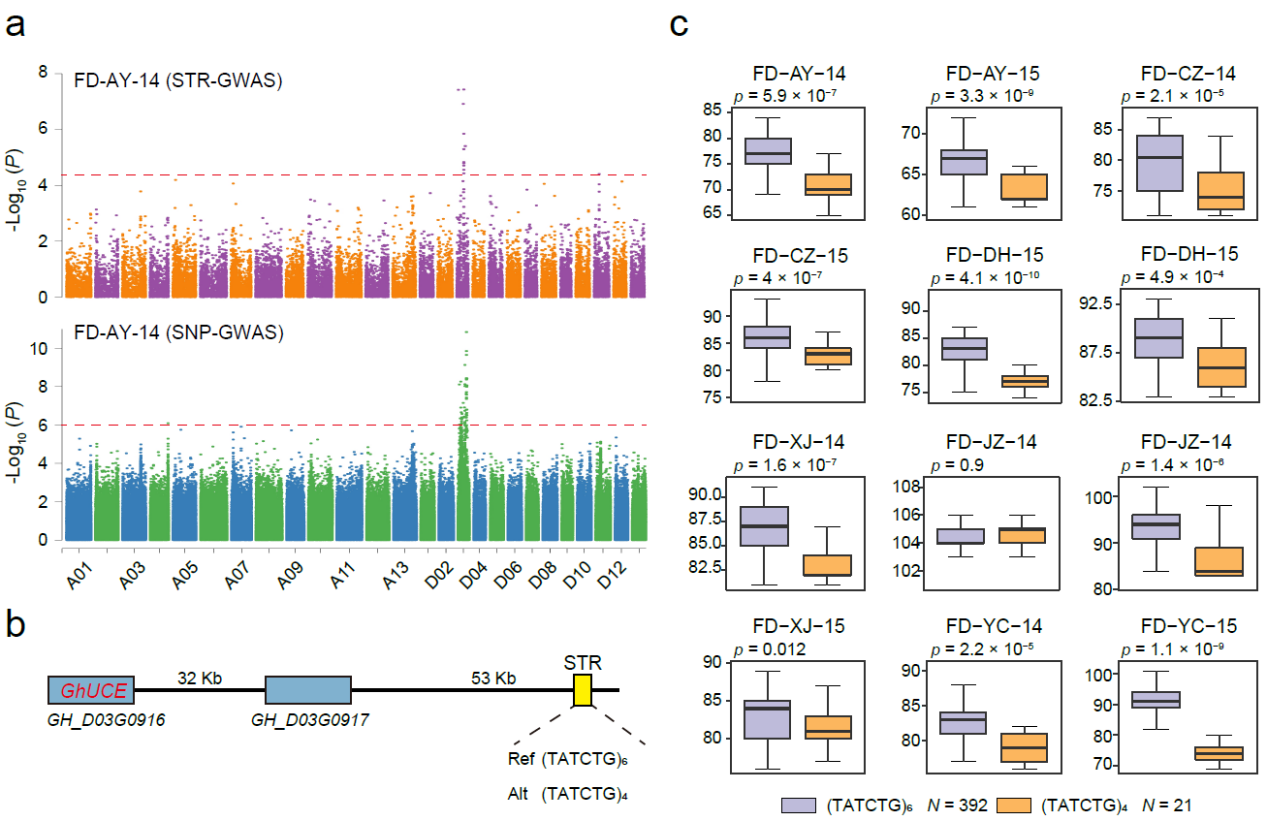


**Figure S13. Association of STR D03:32951122 with days to flower (FD) in P3.**

(**a**) Combined Manhattan plot for FD using STR(Top) and SNP (bottom). (**b**) The Locus Zoom plot (D03: from 32.898 to 32.954 Mb). (**c**) Box plot for FD based on the two haplotypes of D03:32951122 STR (*n* = 392 versus 21) in P3. Center line, median; box limits, upper and lower quartiles; whiskers, 1.5× the interquartile range (**p* < 0.05, two-sided T-test). AY: Anyang in Henan Province; CZ: Cangzhou in Hebei Province; DH: Dunhuang in Gansu Province; JZ: Jingzhou in Hubei Province; XJ: Alaer in the Xinjiang; YC: Yancheng in Jiangsu Province. 14: 2014; 15:2015.


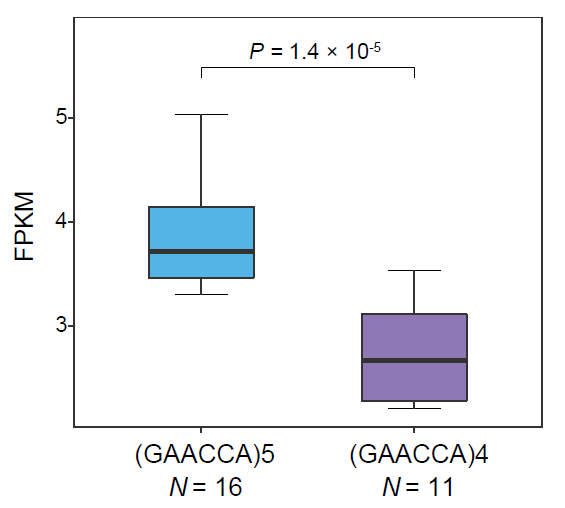


**Figure S14. Box plot for expression of *GH_D06G1697* based on the two haplotypes of** **D06:54211118 STR.**

Center line, median; box limits, upper and lower quartiles; whiskers, 1.5× the interquartile range (Two-sided t-test).


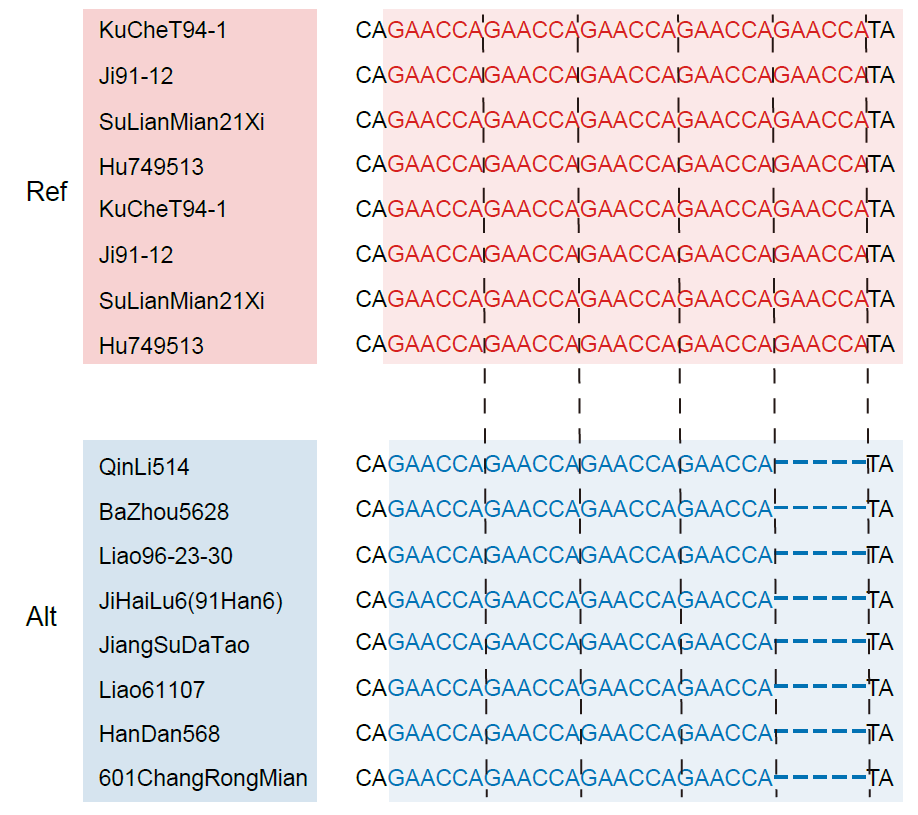


**Figure S15. Polymorphism of STR D06:54211118 for 16 accessions in P1 based on resequencing data.**

Ref, reference type in eight cultivars; Alt, alternate type in another eight cultivars.


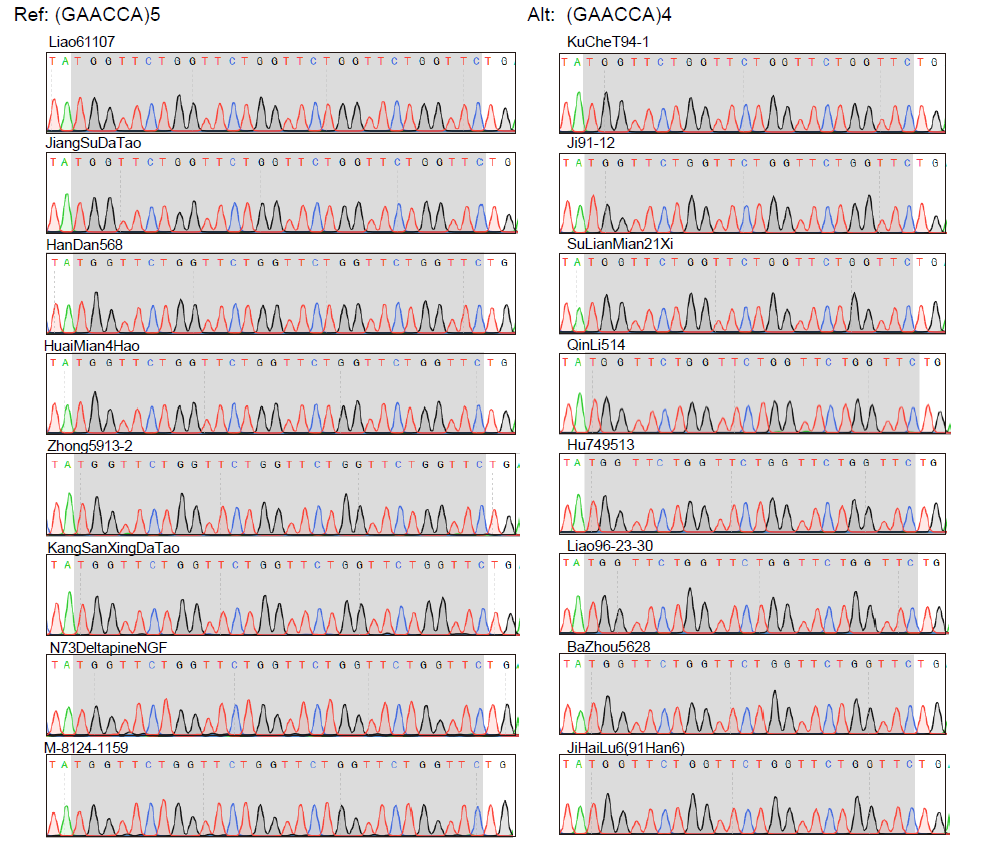


**Figure S16. PCR based verification for the polymorphism of STR D06:54211118 for 16 accessions in P1** **population.**

Ref, reference type in eight cultivars; Alt, alternate type in another eight cultivars.


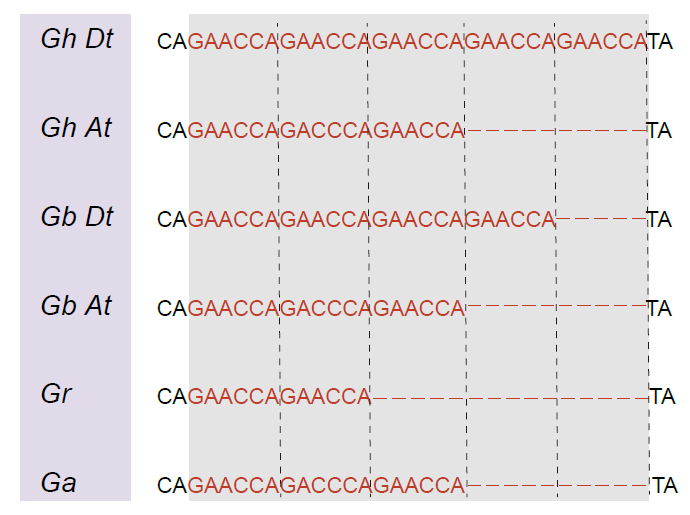


**Figure S17. Genotyping of STR D06:54211118 in *Gossypium* species.**

*Gh* At and *Gh* Dt indicated the A and D subgenome from *G. hirsutum* TM-1, respectively. *Gb* At and *Gb* Dt indicated the A and D subgenome from *G. barbadense* Hai7124, respectively. *Ga*, *G. arboretum*. *Gr,* *G. raimondii.*

*
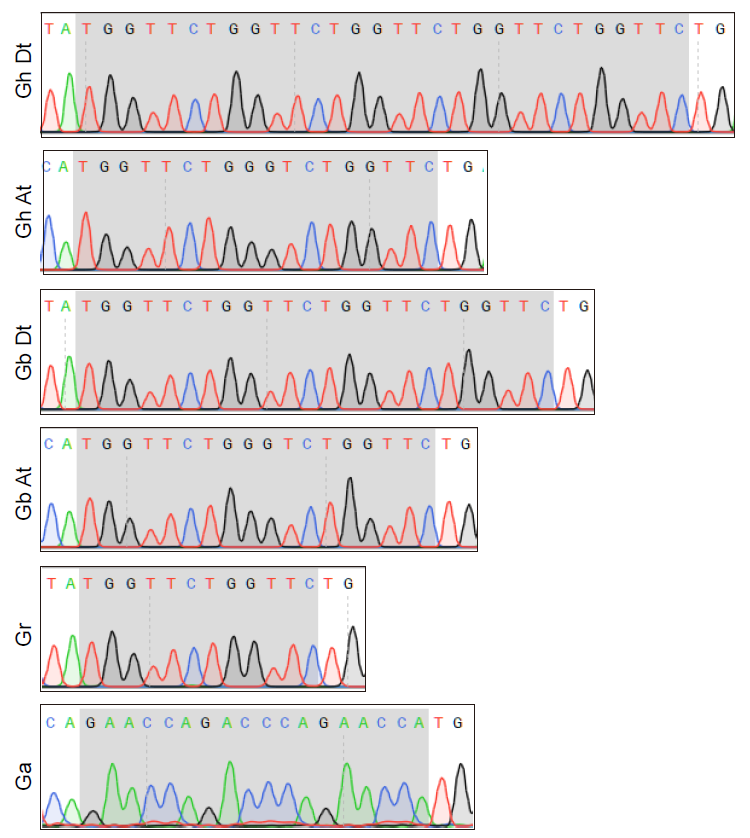
*

**Figure S18. PCR based verification of STR D06:54211118 in *Gossypium* species.** *Gh* At and *Gh* Dt indicated the A and D subgenome from *G. hirsutum* TM-1, respectively. *Gb* At and *Gb* Dt indicated the A and D subgenome from *G. barbadense* Hai7124, respectively. *Ga*, *G. arboretum*. *Gr*, *G. raimondii.*


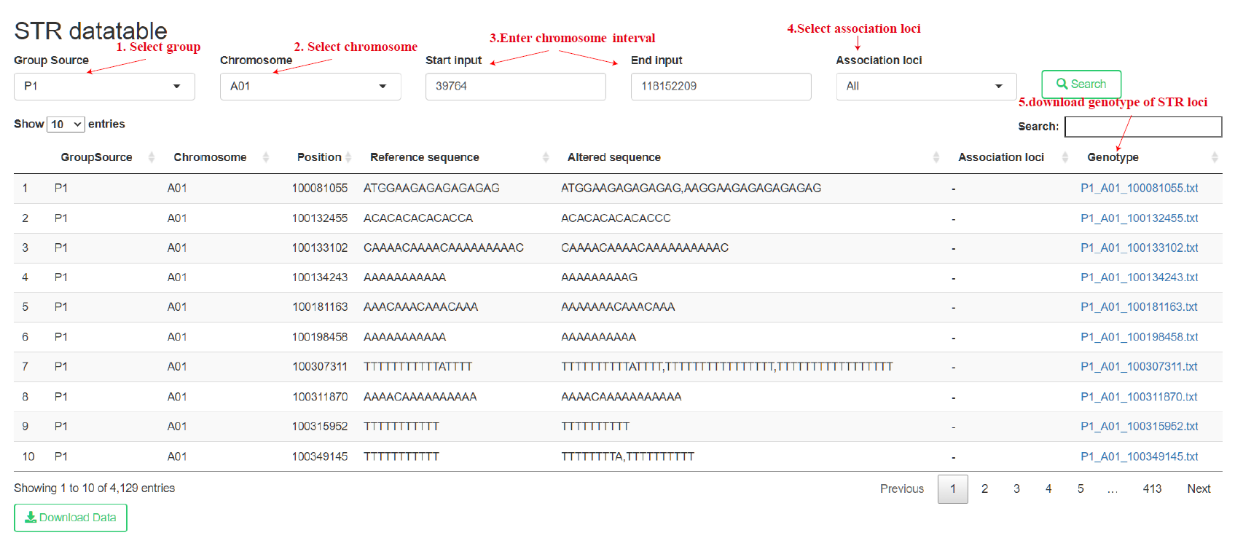


**Figure S19. The user-friendly interface of CottonSTRDB.**

Instructions are as follows: First, choose group source (P1, P2, P3) and the chromosome where the STR is located. Second, input the corresponding chromosome position interval. Click the “Search” button, followed by the “Genotype”, you can download the genotype of specific STR locus. Third, click “Download data” button and you will get the STR data of interest.

**Table S3. Primers sequence for PCR of STR D06:54211118 in this study**

| **Genome or population** | **Subgenome** | **F-primer (5'to3')** | **R-primer (5'to3')** |
| --- | --- | --- | --- |
| *Gh* | Dt | ATTGTGTATTCCTGTTTGGATGGTG | GCTGTGGGATTGAGAAGG |
|  | At | TACGATAAAGCCGAAGATGACGAGC | TGTAAATAAGTCCCTCCCTTGAATA |
| *Gb* | Dt | TACGATAAAGCAGAAGATGATGAGG | CCCTTTGCACATCGTTTCAGTTTCA |
|  | At | TACGATAAAGCCGAAGATGACGAGC | TATAAGTACTTTGCACATTGTTTCA |
| *Gr* | / | GCAAAGGACAATGGAGGCA | GTAGAACTCTTTGATTTTACATAGG |
| *Ga* | / | TCGTGATAGGGATGCTGA | GTGCTGTGGGATTCAGAAGGGGAGG |
| *Gh* poulation | / | ATTGTGTATTCCTGTTTGGATGGTG | GCTGTGGGATTGAGAAGG |

At, Dt: two subgenomes of allotetraploid cotton.

**Table S6. Summary of chromosomal distribution and average density of STRs mapped on *G. hirsutum* chromosomes**

| **Chr.** | **STR number** | **Density**  **(Per Kb)** | **Chr.** | **STR number** | **Density**  **(Per Kb)** |
| --- | --- | --- | --- | --- | --- |
| A01 | 23,482 | 0.203 | D01 | 19,034 | 0.301 |
| A02 | 20,601 | 0.195 | D02 | 19,782 | 0.290 |
| A03 | 22,510 | 0.207 | D03 | 15,623 | 0.297 |
| A04 | 17,054 | 0.199 | D04 | 16,282 | 0.293 |
| A05 | 29,026 | 0.268 | D05 | 22,441 | 0.359 |
| A06 | 24,204 | 0.196 | D06 | 19,293 | 0.302 |
| A07 | 22,360 | 0.237 | D07 | 18,968 | 0.332 |
| A08 | 24,701 | 0.202 | D08 | 21,054 | 0.312 |
| A09 | 20,132 | 0.248 | D09 | 16,624 | 0.327 |
| A10 | 25,060 | 0.223 | D10 | 20,536 | 0.314 |
| A11 | 28,555 | 0.241 | D11 | 22,695 | 0.326 |
| A12 | 24,259 | 0.231 | D12 | 19,503 | 0.324 |
| A13 | 23,294 | 0.216 | D13 | 19,353 | 0.307 |
| At | 305,238 | 0.220 | Dt | 251,188 | 0.314 |
| All | 556,426 | 0.254 |  |  |  |

At, Dt: two subgenomes of allotetraploid cotton *G. hirsutum*.

**Table S14. Enriched transcription factors with exonic STRs in 6,021 genes**

| **Type** | **Gene hits in selected set** | **All genes in selected set** | **Gene hits in background** | **All genes in background** | **Odd ratio** | ***p* value** |
| --- | --- | --- | --- | --- | --- | --- |
| C2H2 | 83 | 6,021 | 356 | 72,761 | 2.82 | 1.78E-14 |
| MYB | 94 | 6,021 | 450 | 72,761 | 2.52 | 1.26E-13 |
| WRKY | 62 | 6,021 | 232 | 72,761 | 3.23 | 2.37E-13 |
| ERF | 93 | 6,021 | 454 | 72,761 | 2.48 | 5.51E-13 |
| Trihelix | 35 | 6,021 | 87 | 72,761 | 4.86 | 4.33E-12 |
| HB | 63 | 6,021 | 312 | 72,761 | 2.44 | 3.84E-09 |
| TCP | 27 | 6,021 | 72 | 72,761 | 4.53 | 3.94E-09 |
| SRS | 13 | 6,021 | 26 | 72,761 | 6.04 | 3.64E-06 |
| bHLH | 61 | 6,021 | 370 | 72,761 | 1.99 | 4.06E-06 |
| AP2 | 18 | 6,021 | 54 | 72,761 | 4.03 | 5.74E-06 |
| OFP | 18 | 6,021 | 55 | 72,761 | 3.95 | 7.08E-06 |
| NAC | 52 | 6,021 | 301 | 72,761 | 2.09 | 7.14E-06 |
| NF-YC | 12 | 6,021 | 28 | 72,761 | 5.18 | 2.88E-05 |
| SBP | 17 | 6,021 | 58 | 72,761 | 3.54 | 4.09E-05 |
| GRF | 12 | 6,021 | 35 | 72,761 | 4.14 | 1.63E-04 |
| C2C2-Dof | 24 | 6,021 | 118 | 72,761 | 2.46 | 2.08E-04 |
| NF-YB | 13 | 6,021 | 48 | 72,761 | 3.27 | 6.02E-04 |
| SAP | 4 | 6,021 | 4 | 72,761 | 12.08 | 1.86E-03 |
| Orphans | 28 | 6,021 | 173 | 72,761 | 1.96 | 1.97E-03 |
| LOB | 23 | 6,021 | 131 | 72,761 | 2.12 | 1.98E-03 |
| C2C2-CO-like | 9 | 6,021 | 30 | 72,761 | 3.63 | 2.28E-03 |
| ARF | 15 | 6,021 | 71 | 72,761 | 2.55 | 3.17E-03 |
| G2-like | 22 | 6,021 | 129 | 72,761 | 2.06 | 3.18E-03 |
| zf-HD | 11 | 6,021 | 47 | 72,761 | 2.83 | 4.08E-03 |
| C3H | 25 | 6,021 | 169 | 72,761 | 1.79 | 9.61E-03 |
| PLATZ | 10 | 6,021 | 47 | 72,761 | 2.57 | 1.05E-02 |
| LFY | 2 | 6,021 | 2 | 72,761 | 12.08 | 3.16E-02 |
| CSD | 4 | 6,021 | 13 | 72,761 | 3.72 | 3.63E-02 |
| GeBP | 4 | 6,021 | 15 | 72,761 | 3.22 | 5.23E-02 |
| C2C2-GATA | 12 | 6,021 | 89 | 72,761 | 1.63 | 1.29E-01 |
| bZIP | 24 | 6,021 | 217 | 72,761 | 1.34 | 1.80E-01 |
| EIL | 3 | 6,021 | 17 | 72,761 | 2.13 | 1.93E-01 |
| Whirly | 1 | 6,021 | 4 | 72,761 | 3.02 | 3.28E-01 |
| HRT | 1 | 6,021 | 5 | 72,761 | 2.42 | 3.79E-01 |
| ARR-B | 5 | 6,021 | 39 | 72,761 | 1.55 | 3.84E-01 |
| E2F-DP | 3 | 6,021 | 23 | 72,761 | 1.58 | 4.46E-01 |
| FAR1 | 3 | 6,021 | 61 | 72,761 | 0.59 | 4.85E-01 |
| RWP-RK | 4 | 6,021 | 35 | 72,761 | 1.38 | 5.38E-01 |
| B3 | 11 | 6,021 | 170 | 72,761 | 0.78 | 5.74E-01 |
| BES1 | 2 | 6,021 | 22 | 72,761 | 1.10 | 7.05E-01 |
| TUB | 2 | 6,021 | 37 | 72,761 | 0.65 | 7.67E-01 |
| Tify | 2 | 6,021 | 38 | 72,761 | 0.64 | 7.67E-01 |
| M-type | 6 | 6,021 | 88 | 72,761 | 0.82 | 8.45E-01 |
| MIKC | 8 | 6,021 | 94 | 72,761 | 1.03 | 8.52E-01 |
| MYB-related | 9 | 6,021 | 126 | 72,761 | 0.86 | 8.71E-01 |
| GRAS | 13 | 6,021 | 156 | 72,761 | 1.01 | 8.85E-01 |
| HSF | 6 | 6,021 | 82 | 72,761 | 0.88 | 1.00E+00 |
| CPP | 2 | 6,021 | 28 | 72,761 | 0.86 | 1.00E+00 |
| BBR-BPC | 1 | 6,021 | 14 | 72,761 | 0.86 | 1.00E+00 |
| CAMTA | 1 | 6,021 | 16 | 72,761 | 0.76 | 1.00E+00 |
| DBB | 1 | 6,021 | 18 | 72,761 | 0.67 | 1.00E+00 |

**Table S15. Flowering genes exhibiting significantly difference according to the genotype of STRs**

| **Gene ID** | **Gene Description** | **STR Position** | **Haplotype association analysis (*p* < 0.05, Student's *t* test)** |
| --- | --- | --- | --- |
| *GH_A02G1005* | Histone-lysine N-methyltransferase ATXR7 | A02:33545168 | PP09nan |
| *GH_A11G1213* | Protein indeterminate-domain 2 | A11:12193879 | PP09an, PP09nan |
| *GH_A11G2131* | Transcription factor MYB102 | A11:46010587 | FD-CZ-15, FD-DH-14, PP09an |
| *GH_D07G2228* | Transcription factor PIF1 | D07:53693437 | PP07nan |
| *GH_D12G1376* | Transcription factor MYB61 | D12:41897050 | PP07nan |
| *GH_A01G0871* | Nuclear transcription factor Y subunit B-3 | A01:13579635 | FD-AY-15, FD-CZ-14, FD-XJ-14 |
| *GH_A05G2327* | Cold shock protein 1 | A05:22958610 | FD-CZ-15 |
| *GH_A05G2491* | Probable sucrose-phosphate synthase 4 | A05:25853406 | FD-CZ-14 |
| *GH_A11G2131* | Transcription factor MYB102 | A11:46010587 | FD-CZ-15, PP09an |
| *GH_D09G1716* | Two-component response regulator-like APRR7 | D09:44158575 | FD-AY-15, FD-CZ-15, FD-DH-14, FD-DH-15, FD-JZ-15, FD-XJ-14, FD-YC-14, FD-YC-15 |
| *GH_D12G2879* | Transcription factor TCP18 | D12:60697094 | FD-AY-15, FD-CZ-15, FD-DH-14, FD-JZ-15, FD-XJ-14, FD-YC-14, FD-YC-15, PP09ku |

**Table S17 Expression of *GH_D06G1697* based on the two haplotypes of D06:54211118 STR.**

| **Code** | **Name** | **Expression of *GH_D06G1697*** | **Genotype of D06:54211118 STR** |
| --- | --- | --- | --- |
| D21 | Zhong5913-2 | 3.717801 | (GAACCA)5 |
| D155 | JiangSuDaTao | 3.714414 | (GAACCA)5 |
| D70 | Lu458 | 5.626841 | (GAACCA)5 |
| D141 | LiaoMian18 | 5.039198 | (GAACCA)5 |
| D233 | JiYuan55(91Han14) | 4.489631 | (GAACCA)5 |
| D81 | JiYuan12-13 | 4.229416 | (GAACCA)5 |
| D232 | ZhongYuanHAS-1 | 4.121938 | (GAACCA)5 |
| D103 | J02-508 | 4.001166 | (GAACCA)5 |
| D202 | SuYuan04-129 | 3.498645 | (GAACCA)5 |
| D203 | Han8901 | 3.660504 | (GAACCA)5 |
| D169 | Yun93Kang393 | 3.351673 | (GAACCA)5 |
| D178 | GK20 | 4.086347 | (GAACCA)5 |
| D184 | DP33B | 3.310206 | (GAACCA)5 |
| D165 | HanDan109 | 3.302222 | (GAACCA)5 |
| D46 | ZhongMianSuo50 | 3.555607 | (GAACCA)5 |
| D181 | GP67 | 3.370492 | (GAACCA)5 |
| D251 | ChangRong67-12 | 2.206969 | (GAACCA)4 |
| D163 | Liao96-23-30 | 3.539956 | (GAACCA)4 |
| D138 | ZhongZi640 | 3.386544 | (GAACCA)4 |
| D41 | KuChe93551 | 3.333198 | (GAACCA)4 |
| D261 | Miscot78-27 | 2.89323 | (GAACCA)4 |
| D167 | Ji91-12 | 2.862907 | (GAACCA)4 |
| D7 | GP93 | 2.665122 | (GAACCA)4 |
| D26 | DaLingMian69Hao | 2.462766 | (GAACCA)4 |
| D59 | MSCO-12 | 2.334277 | (GAACCA)4 |
| D23 | SuLianMian21Xi (91-133) | 2.22491 | (GAACCA)4 |
| D32 | YanCheng1115 | 2.215752 | (GAACCA)4 |
